# Comparing Italian versus European strategies and technologies for respiratory care in NICU: results of a survey of the Union of European Neonatal and Perinatal Societies (UENPS) and the Italian Society of Neonatology (SIN)

**DOI:** 10.1186/s13052-025-01936-6

**Published:** 2025-03-26

**Authors:** Camilla Gizzi, Flavia Petrillo, Maria Luisa Ventura, Luigi Gagliardi, Daniele Trevisanuto, Gianluca Lista, Raffaele Dellacà, Artur Beke, Giuseppe Buonocore, Antonia Charitou, Manuela Cucerea, Boris Filipović-Grčić, Nelly Georgieva Jeckova, Esin Koç, Joana Saldanha, Manuel Sanchez-Luna, Dalia Stoniene, Heili Varendi, Giulia Vertecchi, Luigi Orfeo, Fabio Mosca, Corrado Moretti

**Affiliations:** 1 Division of Neonatology and NICU, Sant’Eugenio Hospital, Rome, ASL RM2 Italy; 2 Division of Pediatrics and Neonatology, Sandro Pertini Hospital, Rome, ASL RM2 Italy; 3 Union of European Neonatal and Perinatal Societies (UENPS), Milan, Italy; 4 Maternal and Child Department ASL Bari, Ospedale Di Venere, Bari, Italy; 5 Neonatal Intensive Care Unit, Fondazione IRCCS San Gerardo dei Tintori, Monza, Italy; 6 Division of Neonatology and Pediatrics, Ospedale Versilia, Azienda USL Toscana Nord Ovest, Viareggio, Pisa, Italy; 7 Department of Woman’s and Child’s Health, University of Padova, Padova, Italy; 8 Division of Pediatrics, Neonatal Intensive Care Unit and Neonatology, “V.Buzzi” Ospedale dei Bambini ASST -FBF- Sacco, Milan, Italy; 9 TechRes Lab, Department of Electronics, Information and Biomedical Engineering (DEIB), Politecnico di Milano University, Milan, Italy; 10 1st Department of Obstetrics and Gynecology, Semmelweis University, Budapest, Hungary; 11 Department of Pediatrics, Università degli Studi di Siena, Siena, Italy; 12 Department of Pediatrics, Rea Maternity Hospital, Athens, Greece; 13 Neonatology Department, University of Medicine Pharmacy Sciences and Technology “George Emil Palade”, Târgu Mures, Romania; 14 Department of Pediatrics, University of Zagreb School of Medicine, Zagreb, HR Croatia; 15 Department of Pediatrics, University Hospital “Majchin dom”, Sofia, Bulgaria; 16 Division of Neonatology, Department of Pediatrics, School of Medicine, Gazi University, Ankara, Turkey; 17 Department of Pediatrics, Neonatology Division, Hospital Beatriz Ângelo, Loures, Portugal; 18 Neonatology Division, Department of Pediatrics, Hospital General Universitario “Gregorio Marañón”, Madrid, Spain; 19 Department of Pediatrics, Lithuanian University of Health Sciences, Kaunas, Lithuania; 20 Department of Paediatrics, University of Tartu, Tartu University Hospital, Tartu, Estonia; 21 SIN “Società Italiana di Neonatologia”, Ospedale Isola Tiberina Gemelli Isola, Rome, Italy; 22 Department of Pediatrics, Department of Clinical Sciences and Community Health, Fondazione IRCCS Cà Granda Ospedale Maggiore Policlinico Milan, University of Milan, Milan, Italy; 23 Department of Pediatrics, Policlinico Umberto I, Sapienza University of Rome, Rome, Italy

**Keywords:** Mechanical ventilation, Delivery room, Preterm infants, RDS, Surfactant, Survey, Caffeine, Steroids

## Abstract

**Background:**

Our survey aimed to compare information on respiratory care in Neonatal Intensive Care Units (NICUs) in Italy and in the European and Mediterranean region.

**Methods:**

Cross-sectional electronic survey. An 89-item questionnaire focusing on the current modes, devices, and strategies employed in neonatal units in the domain of respiratory care was sent to directors/heads of 528 NICUs.

**Results:**

The response rate was 75% (397/528 units). The median number of NICU beds and the admission rate per unit/year of preterm infants < 1500 g was significantly lower in Italy compared with Europe (*p* < 0.001). In most Italian Delivery Rooms (DR) full resuscitation is given from 22 to 23 weeks gestational age, while 21.0% of the European units initiate from 24 weeks. Initial FiO_2_ is set as per American Academy of Pediatrics guidelines in 81.1% of Italian units compared to 30.9% of the European ones (*p* < 0.001). DR surfactant is less often given through Less-Invasive-Surfactant-Administration (LISA) in Italy (53.4% vs. 76.2% of units, *p* < 0.03). Volume-targeted, synchronized intermittent positive-pressure ventilation (IPPV) is the preferred invasive mechanical ventilation (MV) mode to treat acute RDS across the surveyed units, however 22.9% % of Italian centers vs. 6.8% of the European ones use HFOV as first choice (*p* < 0.001). During HFOV, 78% of Italian NICUs set mean airway pressure (MAP) following a lung recruitment procedure compared to 41% of the centers in Europe (*p* < 0.001). In the NICUs, most of the non-invasive (NIV) modes used are nasal CPAP and nasal IPPV. For infants on NIV, LISA strategy is used in 25.6% of Italian vs. 60.0% of European units (*p* < 0.001). 70% of surveyed units use a brand caffeine. Inhaled steroids are used in 42.3% of Italian vs. 65.4% of European NICUs (*p* < 0.001).

**Conclusions:**

respiratory support strategies among the surveyed Italian and European NICUs are quite dissimilar in some areas, particularly where high-quality evidence is lacking. We believe that hese data will allow stakeholders to make comparisons and to identify opportunities for improvement.

**Supplementary Information:**

The online version contains supplementary material available at 10.1186/s13052-025-01936-6.

## Introduction

Respiratory care in Neonatal Intensive Care Units (NICU) has a significant impact on neonatal survival and outcomes. In recent years, novel technologies and strategies for respiratory support have been introduced in neonatology with the aim of protecting the developing lungs of ventilated infants. Indeed, preventing the use, or shortening the length, of invasive mechanical ventilation (MV) significantly decrease the risk of bronchopulmonary dysplasia (BPD) and neurodevelopmental impairment (NDI) [1, 2]. In the context of conventional invasive MV, the volume-targeted ventilation (VTV) or volume guarantee (VG) seems to highly impact outcomes like BPD or severe intraventricular haemorrhage (IVH) [3]. Similarly, High Frequency Oscillatory Ventilation (HFOV) coupled with VG seems to be more effective in maintaining pCO_2_ levels within the target range [4, 5] and may also reduce proinflammatory systemic reactions, length of ventilation [6] and BPD [7], although there is no equally solid evidence in the literature on its benefits. In the context of non-invasive ventilation (NIV) advances have been made in improving technologies to provide nasal continuous positive airway pressure (NCPAP), as the innovative jet systems [8] and to synchronize nasal intermittent positive pressure ventilation (NIPPV) to the infant’s spontaneous breathing [9], making these modes more effective. Finally, among strategies, the novel surfactant administration techniques coupled with NIV and early caffeine are able to further reduce the number of infants requiring invasive MV [10, 11]. Nevertheless, several studies report that is difficult to translate research results into actual clinical practice. Neonatal units often do not base their choices on the evidence coming from large randomized controlled trials (RCT) [12], indicating that there is a need for more effective methods to ensure evidence-based practice [13]. The above-mentioned considerations prompted the Union of European Neonatal and Perinatal Societies (UENPS) and the Pulmonology Board of the Italian Society of Neonatology (SIN) to perform a European survey with the aim of comparing practices of neonatal respiratory support in NICUs in Italy and in Europe, and evaluating its relationship with the evidence from the literature. A better understanding of the variations in practice between neonatal networks can help identify best practices and opportunities for improvement.

## Materials and methods

This is a large cross-sectional electronic survey entitled “European survey on neonatal respiratory care in NICUs”. The questionnaire was developed by a committee of experts on neonatal pneumology gathered from the Pulmonology Board of the Italian Society of Neonatology and the UENPS Scientific Board and designed as web-based following the Checklist for Reporting Results of Internet E-Surveys (CHERRIES) guidelines [14]. It was composed of 89 questions related to neonatal respiratory management (questionnaire available as Additional file 1). The survey addressed five domains: (A) general information, (B) the modes, devices and strategies employed in the Delivery Room (DR); (C) the modes, devices and strategies employed in the NICU; (D) the drugs used in NICU for neonatal respiratory diseases; (E) the mechanical ventilators available in NICU. The items included multiple-choice, fill-in, and ‘yes/no’ questions. For distribution purposes, the Presidents or Secretaries of all National Neonatal Societies in Europe were emailed by the study coordinator on behalf of the president of UENPS, requesting the list of directors of NICUs at the national level. The directors subsequently received an email invitation containing a unique access link to the web-based survey, powered by SurveyMonkey **®** (San Mateo, CA, USA). Participants were informed that all responses would be anonymized and encrypted before analysis. Completion of the survey encompassed the informed consent for the respondents’ participation. A reminder was sent to non-responders every 3 weeks, for a maximum of 4 times. After that, non-responders were registered as such. In those countries with a restricted contact information policy (France, Germany, Spain, the Netherlands, Switzerland, Slovakia, Serbia, Croatia, Montenegro, Belgium, Austria, Denmark), the invitation to participate in the Survey was distributed at national level through the National Society’s mailing list. Directors who accepted to participate gave their consent by writing their email address on an online contact form set up on purpose. Participation was completely voluntary. The survey was first sent in February 2022 and closed by July 2022. The study was submitted to the Ethics Committee of the Azienda Ospedale Università di Padova, which reviewed it and awarded an exemption letter (protocol n. 396n/AO/23), as it did not meet criteria for human-subject research. Research was carried out in line with the principles of the Declaration of Helsinki.

### Statistical analysis

The completed questionnaires were reviewed by two independent investigators to avoid technical errors (e.g., duplications). All the data were examined via descriptive analysis. Categorical data were expressed as numbers and percentages while continuous data were expressed as median and interquartile range (IQR). The comparisons between Italy and Europe were performed using Kruskal Wallis distribution-free analysis of variance (for continuous variables) or Chi-square (χ^2^) tests (for categorical variables), as appropriate. No post hoc multiple comparisons between groups were performed. Statistical analysis was performed using the Stata 15 statistical Package (StataCorp, College Station, TX, USA).

## Results

**Fig. 1:**
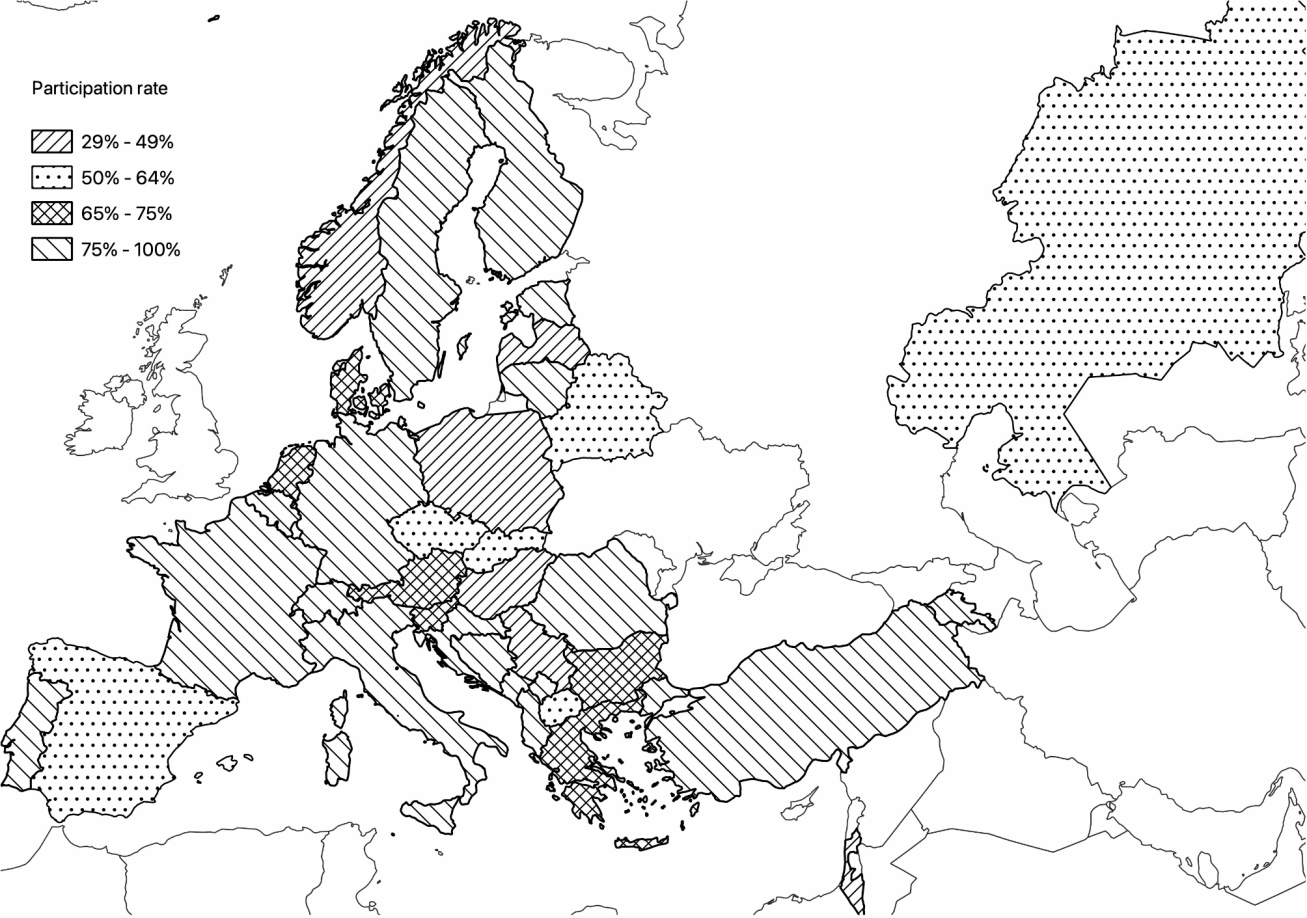
Distribution of the surveyed centres

Five-hundred and twenty-eight NICUs across 37countries in the European and Mediterranean geographic area were contacted (Fig. 1). The United Kingdom and Ireland were not involved in this study, as their scientific societies did not respond to our invitation. The overall response rate was 75%: out of 528 units contacted, 410 responded; 13 of these were not NICUs and were excluded from further analysis. At closure, 397 NICUs were included in the study, 94 in Italy and 303 in the rest of Europe (list available as Additional File 2). Ninety-seven out of the 397 participating NICUs (24.4%) did not belong to European Union (EU) countries. The Italian response rate was 81.7% (94/115).

### General information

In Italy, less than half (34.7%) of the responding units were academic hospitals, while 79.0% of the European participating centers were academic (*p* < 0.001). The median (IQR) number of NICU beds in Italy was 8 (6-10) compared with 15 (10-23) in Europe (*p* < 0.0001). Consequently, the admission rate per unit per year of preterm infants weighing < 1500 g was statistically different in Italy compared with Europe, reflecting different policies on perinatal care regionalization: in Italy 40.2% of NICUs admit < 30 VLBW infants per year; 38.5% 30–50; 20.6% 50–100; and 1.1% >100, compared to 19.3%, 24.7%, 30.4%, and 25.5% respectively (*p* < 0.001). The patient/nurse ratio is 2:1 in 44.1% of Italian NICUs, 3:1 in 39.8%, and 4:1 in 14.0%. These percentages changes in Europe in 28.4% s, 31.0%, and 16.2%, respectively (*p* < 0.001). General information was also compared between academic and non-academic hospitals in Italy and in Europe. In Italy, the median (IQR) number of NICU beds was 10 (6.5–15) in academic centres and 6 (5-9) in non-academic ones (*p* = 0.0001), and the admission rate of VLBW infants per year was < 30 in 12.9%; 30–50 in 41.9%; 50–100 in 41.9%; and > 100 in 3.2% of academic units, compared to 54.1%, 36.1%, 9.8%, and 0.0% of non-academic units, respectively (*p* = 0.000). In Europe, the median (IQR) number of NICU beds was 15 (10-24) in academic centres and 12 (8-17) in non-academic ones (*p* = 0.0003), and the admission rate of VLBW infants per year was < 30 in 15.7%; 30–50 in 23.8%; 50–100 in 31.9%; and > 100 in 28.5% of academic units, compared to 32.7%, 27.9%, 24.6%, and 14.7% of non-academic units, respectively (*p* = 0.007).

National face-to-face courses on neonatal ventilation are the most attended way to upgrade training on respiratory care and techniques in Italy and in Europe.

### The modes, devices and strategies employed in the delivery room

More than 90% of all responding units were birth centers. Delivery Room technologies and strategies are not dissimilar among Italian and European units. The lowest gestational age (GA) at which full resuscitation is initiated is 22–23 weeks in 94.5% of the Italian units, while 21.0% of the European units initiate from 24 weeks. Comparing EU and non-EU countries, 23.1% of the EU NICUs responded to this question 22 weeks; 52.7% 23 weeks; 17.9% 24 weeks; 1.5% 25 weeks; and 0.4% 26 weeks, versus 45.5%, 26.7%, 10.4%, 1.2%, and 2.3% of the non-EU NICUs, respectively (*p* = 0.000). A T-piece resuscitator is available in about 90% of all responding units, while a mechanical ventilator is available in 41.1% of the Italian and in 54.3% of the European DRs. The device routinely used for respiratory stabilization in non-invasive mode is the T-piece ventilator, used in about 70% of all responding units. The new R-Pap system [15] has been adopted in about 9% of Italian and European DRs. In Italy no centers routinely utilize a self-inflating bag, compared to 3.7% of the European centers. Only 10% of units across Europe use a mechanical ventilator for DR stabilization. This strategy is more frequently used among European academic units compared to non-academic ones (13.0% vs. 3.8%, *p* = 0.028). Facial masks (about 36%), nasal masks (about 17%), and short binasal prongs (about 34%) as first choice for respiratory stabilization in non-invasive mode are used in a similar percentage of centers in Italy and in Europe. When setting initial FiO_2_, the American Academy of Pediatrics or the European Resuscitation Council guidelines for neonatal resuscitation are followed by 81.1% and 17.8% of units in Italy and by 30.9% and 59.1% in Europe, respectively (*p* < 0.001). When comparing EU and non-EU centers, the American Academy of Pediatrics and European Resuscitation Council guidelines for neonatal resuscitation are followed by 37.7% and 54.9% of EU units, respectively, and by 61.6% and 29.1% of non-EU units (*p* = 0.000). No significant differences were observed between the number of Italian and European centers that aim at the saturation target of 80–85% at 5 min of life for preterm infants < 28 weeks’ gestation and that use heated and humidified gases for resuscitation, being about 80% in both groups. Table 1 describes stabilization strategies at birth for spontaneously breathing 23^+ 0^- 24^+ 6^ and 25^+ 0^- 27^+ 6^ weekers, pulmonary recruitment manoeuvres used during stabilization, and surfactant administration strategies used in the DR. Surfactant is given in the DR in 78.9% of Italian participating units compared to 88.5% of European ones (*p* = 0.023). An ECG monitor is available in about 70% of the responding centers in Italy compared to 60% in Europe (*p* = 0.041). End-tidal CO_2_ detectors are slightly less used in Italy (20.0% vs. 29.3%, *p* = 0.79), while respiratory function monitors (RFM) are more widespread (26.7% vs. 18.6%, *p* = 0.037). Finally, DR caffeine is given in about 70% of the units, regardless of geographical area.

**Table 1:** Respiratory stabilization strategies in the delivery room

**Respiratory stabilization strategy at birth for a spontaneously breathing 23** ^**+ 0**^ **- 24** ^**+ 6**^ **weeker**			
	Italy (n.90)*	Europe (n.269)**	**p**
NCPAP	35 (38.9)	108 (40.1)	
NIPPV	19 (21.1)	51 (19.0)	
Synchronized NIPPV	6 (6.7)	15 (5.6)	
NBiPAP	4 (4.4)	14 (5.2)	
Nasal High Flow Therapy (nHFT)	0 (0.0)	2 (0.7)	
Invasive ventilation	26 (28.9)	74 (27.5)	
Resuscitation not initiated at these GA	0 (0.0)	5 (1.8)	
*p* = 0.835			
***Respiratory stabilization strategy at birth for a spontaneously breathing 25*** ^***+ 0***^ ***- 27*** ^***+ 6***^ ***weeker***			
NCPAP	57 (63.3)	178 (66.2)	
NIPPV	21 (23.3)	44 (16.4)	
Synchronized NIPPV	5 (5.6)	16 (5.9)	
NBiPAP	6 (6.7)	21 (7.8)	
Nasal High Flow Therapy (nHFT)	0 (0.0)	2 (0.7)	
Invasive ventilation	1 (1.1)	8 (3.0)	
*p* = 0.603			
***Pulmonary recruitment manoeuvre used during stabilization of preterm infants***			
None	27 (30.0)	85 (31.6)	
Sustained Lung Inflation	4 (4.4)	40 (14.9)	
Incremental CPAP/PEEP trial	33 (36.7)	88 (32.7)	
Incremental/decremental CPAP/PEEP trial	24 (26.7)	52 (19.3)	
• Initial CPAP/PEEP level (cmH_2_O)	5.4 (0.8)	5.4 (0.9)	0.84
• Max CPAP/PEEP level (cmH_2_O)	8.0 (1.0)	8.3 (1.1)	0.18
Decremental CPAP/PEEP trial	2 (2.2)	4 (1.5)	
Other	0 (0.0)	0 (0.0)	
*p* = 0.082			
***CPAP/PEEP level set during non-invasive ventilation (NIV) for stabilization***			
CPAP/PEEP (cmH_2_O)	5.8 (0.7)	5.9 (1.0)	0.45
***Delivery Room surfactant administration strategies (multiple choice)***			
	**Italy (n.73)°**	**Europe (n.240)°°**	
Conventional mode (= intubation + MV)	32 (43.8)	164 (68.3)	0.002
INSURE	59 (80.8)	174 (72.5)	0.24
INRECSURE	22 (30.1)	10 (4.2)	< 0.001
LISA /MIST	39 (53.4)	183 (76.2)	0.03
Laryngeal mask	1 (1.4)	3 (1.2)	0.93
Pharyngeal instillation	0 (0.0)	2 (0.8)	0.44

Data are expressed as number (%), initial and max CPAP/PEEP pressure levels are expressed as mean (SD); *ITA responding units 90/94 (95.7%); **EU responding units 269/303 (88.8%); °ITA responding units 73/94 (77.6%); °°EU responding units 240/303 (79.2%). See also text. NCPAP = Nasal Continuous Positive Airway Pressure; NIPPV = Nasal Intermittent Positive Pressure Ventilation; NBiPAP = Nasal Bilevel Positive Airway Pressure; PEEP = Positive End Expiratory Pressure; INSURE = INtubation-SURfactant-Extubation; INRECSURE = INtubation– RECruitment of the lung -SURfactant– Extubation; LISA/MIST = Less Invasive Surfactant Administration/Minimally Invasive Surfactant Therapy

### The modes, devices and strategies employed in the NICU

**Fig. 2:**
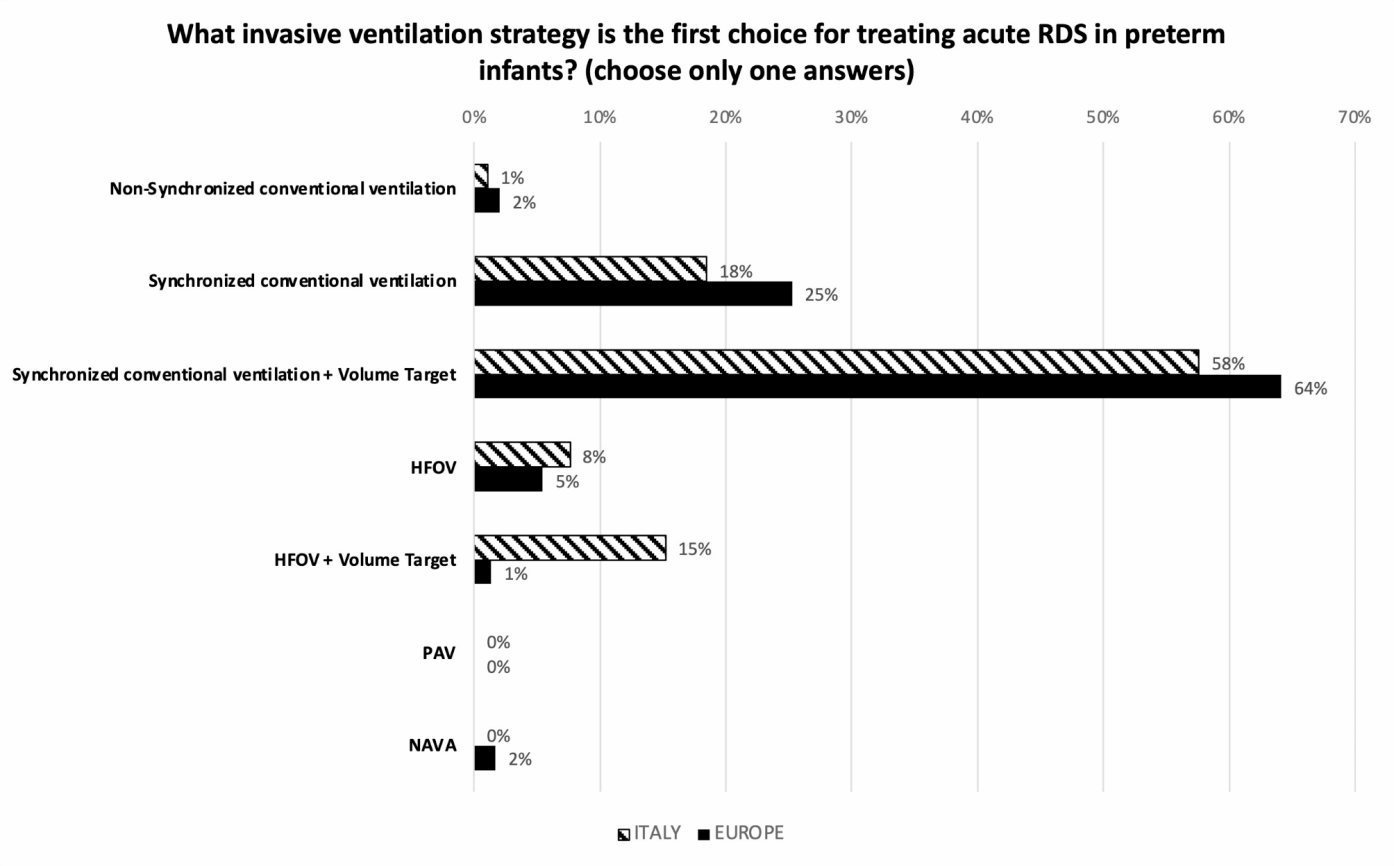
First choice invasive ventilation strategy for treating acute RDS in preterm infants. HFOV = High Frequency Oscillatory Ventilation; PAV = Proportional Assist Ventilation; NAVA = Neurally Adjusted Ventilatory Assist. Numbers express the percentage of responding centers

**Fig. 3:**
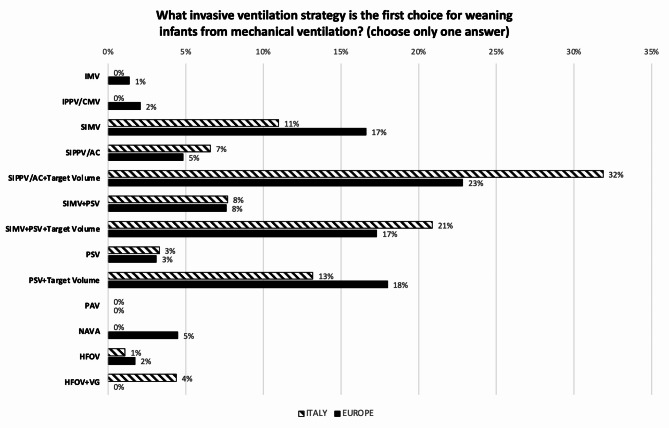
First choice invasive ventilation strategy for weaning preterm infants from mechanical ventilation. IMV = Intermittent Mandatory Ventilation; IPPV/CMV = Intermittent Positive Pressure Ventilation/Continuous Mandatory Ventilation; SIMV = Synchronized Intermittent Mandatory Ventilation; SIPPV/AC = Synchronized Intermittent Positive Ventilation/Assist-Control; PSV = Pressure Support Ventilation; PAV = Proportional Assist Ventilation; NAVA = Neurally Adjusted Ventilatory Assist; HFOV = High Frequency Oscillatory Ventilation; VG = Volume Guarantee. Numbers express the percentage of responding centers

**Fig. 4:**
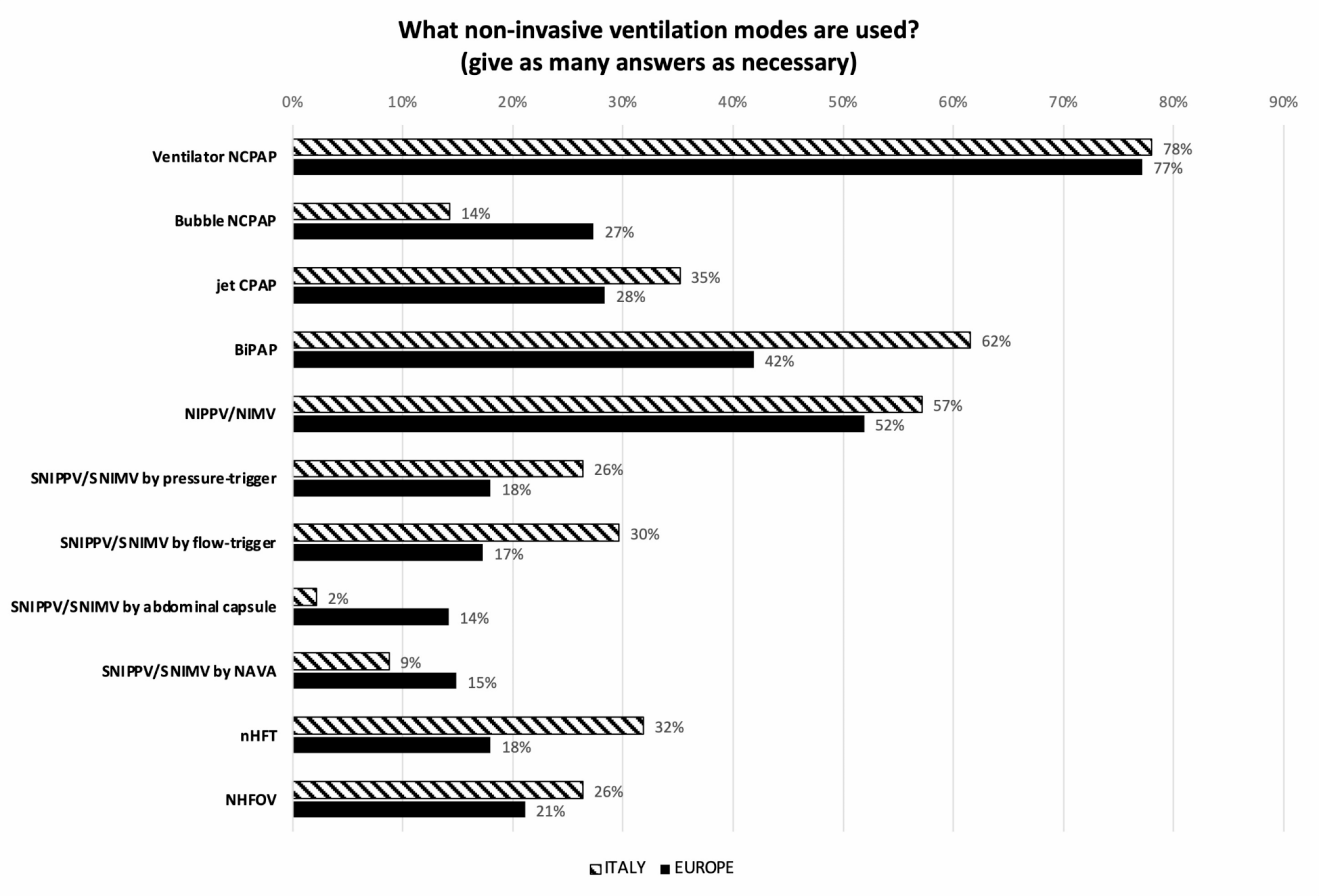
Non-invasive ventilation modes used in NICU. NCPAP = Nasal Continuous Positive Airway Pressure; BiPAP = Bilevel Positive Airway Pressure; NIPPV/NIMV = Nasal Intermittent Positive Pressure Ventilation/Nasal Intermittent Mandatory Ventilation; SNIPPV/SNIMV = Synchronized Nasal Intermittent Positive Pressure Ventilation/Synchronized Nasal Intermittent Mandatory Ventilation; NAVA = Neurally Adjusted Ventilatory Assist; nHFT = nasal High Flow Therapy; NHFOV = Nasal High Frequency Oscillatory Ventilation. Numbers express the percentage of responding centers

Figure 2 compares the invasive ventilation strategies used as first choice for treating acute RDS in preterm infants, in Italy and in Europe. Synchronized intermittent positive pressure ventilation (SIPPV) + VG is largely the favourite strategy. However, 22.9% % of Italian centers use HFOV, with or without VG, as first choice compared to 6.8% of centers across Europe (*p* < 0.001). In this regard, 13.1% of the EU NICUs use HFOV, with or without VG, as first-choice strategy to treat RDS, compared to 3.2% of the non-EU NICUs (*p* = 0.05). The first-choice strategy is different when treating high-risk infants (i.e. extremely low GA infants, preterm premature rupture of membranes (PPROM) with lung hypoplasia, chorioamnionitis, premature birth without antenatal steroid prophylaxis, etc.) in about half (44.6%) of the Italian centers, while 72.0% of the European centers remain on it (*p* = 0.004). The shift for centers who change, is from conventional mechanical ventilation (CMV) towards HFOV or HFOV + VG. While the infant is on CMV, inspiratory time (Ti) is set looking at the flow signal in 62.0% of Italian responding NICUs and depending on the infant’s GA in 35.8%, while in Europe the percentages are 42.0% and 52.6%, respectively (*p* = 0.003). During CMV, positive end expiratory pressure (PEEP) is adjusted by increasing or decreasing its level according to patient’s conditions in 64.1% of Italian versus 79.5% of European NICUs, while a dynamic PEEP (stepwise PEEP increments followed by decrements) is used in 35.9% and 20.5% of units, respectively (*p* = 0.003). During HFOV, 78% of Italian NICUs set mean airway pressure (MAP) following a lung recruitment procedure (stepwise increments followed by decrements) compared to 41% of the centers in Europe (*p* < 0.001). A ‘closed loop’ oxygen control is used in about 30% of all responding units. Nitric Oxide (NO) is available in 88.0% of NICUs in Italy compared to 72.7% in Europe (*p* < 0.003). Figure 3 compares the invasive ventilation strategies used as first-choice for weaning infants from MV, in Italy and in Europe. Moving to non-invasive ventilation, Fig. 4 shows the modes used in Italian and European units. NCPAP is the main used mode, followed by NIPPV. Indeed, 53,9% of Italian and 57,8% of European NICUs preferentially use NCPAP. Among NCPAP pressure generators, jet systems are available in more than 30% of responding units, however these are selected as first choice to deliver CPAP in less than 20%, both in Italy and in Europe. NIPPV, synchronized and non-synchronized, is considered as the main non-invasive mode in 33.0% of Italian vs. 28.7% of European centers (*p* = 0.165). In European academic units, NIPPV is more widely used, while nasal High Flow Therapy (nHFT) is less frequently employed as the main non-invasive mode compared to non-academic units (18.8% vs. 3.3% and 8.3% vs. 0.9%, respectively; *p* = 0.006). Non-invasive Ventilation– Neurally Adjusted Ventilatory Assist (NIV-NAVA) and nasal HFOV (NHFOV) are used in less than 15% and 30% respectively, in Italian and European units. No differences were seen regarding the availability of non-invasive interfaces: almost all NICUs have short binasal prongs and nasal masks, and about 40% also use double-inspiratory loop cannulas (DILC). About 60% of all responding NICUs has no protocols to move from invasive to non-invasive ventilation and vice versa. Extracorporeal Membrane Oxygenation (ECMO) is available in 14.2% of responding hospitals in Italy compared to 27.4% in Europe. Only 10.9% of Italian and 5.2% of European NICUs have a standardized protocol for performing a tracheostomy in chronically ventilated preterm infant. Finally, the incidence [median (IQR) of cases per year] of severe BPD (i.e. need for oxygen and NIV or MV at 36 weeks post-conceptional age) in infants with GA < 29 weeks is similar in Italy and Europe: 10 (2-4) and 11 (5-23) cases per year, respectively. However, there are some significant differences regarding the incidence of BPD between academic and non-academic centers, both in Italy and Europe. Specifically, in Italy, the reported incidence of severe BPD is 19 (10-23) cases per year in academic centers and 7 (2-19) in non-academic centers (*p* = 0.0053). In Europe, the difference is also statistically significant, with the median incidence of severe BPD ranging from 13 (5-25) cases per year in academic centers to 8.5 (1-20) in non-academic centers (*p* = 0.0137). This observation likely reflects the higher prevalence of obstetric pathology, and consequently of more complex neonatal cases, in academic centers.

### The drugs used in NICU for neonatal respiratory diseases

#### Surfactant

**Fig. 5:**
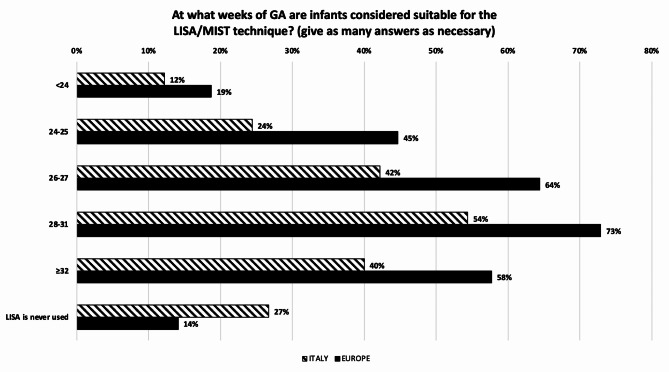
Gestational ages at what infants are considered suitable for the LISA/MIST technique. GA = Gestational Age; LISA/MIST = Less Invasive Surfactant Administration/Minimally Invasive Surfactant Therapy. Numbers express the percentage of responding centers

**Fig. 6:**
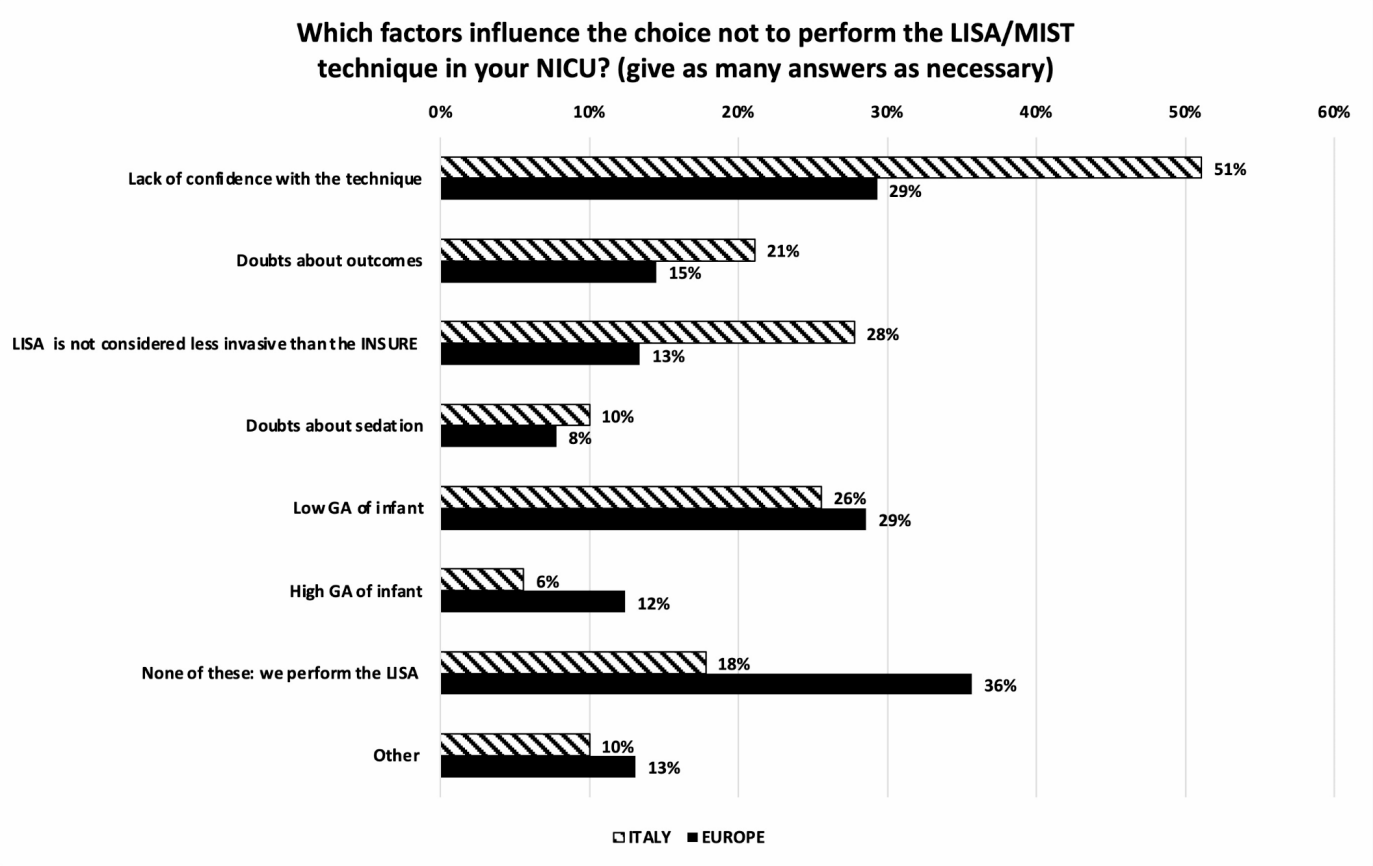
Factors influencing the choice not to perform the LISA/MIST technique in NICU. LISA/MIST = Less Invasive Surfactant Administration/Minimally Invasive Surfactant Therapy. Numbers express the percentage of responding centers

The ranking of parameters considered as the main indicator of surfactant need for an infant on non-invasive ventilation in Italy is: (1) FiO_2_; (2) SpO_2_/FiO_2_; (3) Chest X-Ray; (4) LUS (lung ultrasound); and (5) Silvermann score. In Europe is: (1) FiO_2_; (2) SpO_2_/FiO_2_; (3) Chest X-Ray; (4) Silvermann score; and (5) LUS. The FiO_2_ threshold to administer surfactant followed in Italy is 0.25–0.30 in 58.2% and 0.31–0.4 in 35.2% of the NICUs, in Europe the percentage changes to 44.9% and 31.8%, respectively (*p* = 0.097). In Italy and in Europe almost all NICUs use porcine surfactant, giving an initial dose of 200 mg/kg. When administering surfactant, the relation between vials and dosage is managed by giving the correct dose/kg regardless of how many vials must be opened in 82.2% of units in Italy and in 60.7% in Europe (*p* < 0.001), the remaining units adjust the dosage in order either to use up the surfactant in the vial (increasing the dose) or to avoid opening a new vial (decreasing the dose). In Italy the Less Invasive Surfactant Administration (LISA) or Minimally Invasive Surfactant Therapy (MIST) strategy are used in 25,6% of units, compared to 60.0% of units in Europe (*p* < 0.001). To perform LISA, 86.9% of Italian NICUs use a purpose-built surfactant instillation catheter, compared to 67.6% of European ones. Gestational age at which infants are considered suitable for the LISA/MIST procedure are depicted in Fig. 5. In Italy, only 20.0% of units do not perform a lung recruitment manoeuvre before administering surfactant, compared to 43,6% of European units. The performed lung recruiting manoeuvres are: increasing the level of CPAP or PEEP during NIV, before INtubation-SURfactant-Extubation (INSURE) or LISA/MIST technique; optimizing MAP during HFOV, in course of INRECSURE (INtubation– RECruitment of the lung -SURfactant– Extubation) technique; and increasing the level of PEEP (CMV) or MAP (HFOV) during invasive ventilation when extubation is not expected. The INRECSURE strategy is most popular in Italy than in Europe, being used in 42.2% vs. 11.3% of units, respectively (*p* < 0.001). When administering surfactant through an endotracheal tube, a closed circuit is used in 57.8% of units in Italy, compared to 49.3% in Europe (*p* = 0.161). Among Italian and European NICUS, surfactant spreading immediately after endotracheal tube administration, when extubation is expected, is respectively supported by manual PPV in 33.3% vs. 23.9% of the units; by a T-piece resuscitator in 25.5% vs. 21.8%; by ventilator PPV in 25.5% vs. 30.3%; and not supported by PPV if the infant is spontaneously breathing on CPAP with good respiratory drive in 15.6% vs. 23.9% (*p* = 0.143). When using the INSURE technique, the endotracheal tube is removed immediately after surfactant administration in 61.1% of the NICUs in Italy and in 54.2% in Europe, while time is variable in 15.6% and 26.4% of units, respectively. Besides RDS, surfactant is administered across Europe for the following respiratory diseases: transient tachypnoea of the newborn, meconium aspiration syndrome, pneumonia, pulmonary haemorrhage, and neonatal ARDS. Despite controversial, about 27% of Italian and European NICUs use surfactant to treat congenital diaphragmatic hernia. To conclude on surfactant, no analgesic pharmacological and non-pharmacological pre-treatment is given to infants before the INSURE or INRECSURE techniques in 12.2% of the Italian centers versus 21.6% of the European ones (*p* = 0.08). The percentages of no treatment increase up to 24.4% and 29.7% before the LISA approach, respectively. Factors that influence the choice not to perform the LISA/MIST technique in the responding NICUs are shown in Fig. 6. Across Italy and Europe, use on LISA/MIST technique would be increased if dedicated training, more information on outcomes, more scientific publications and more follow-up data on the technique were available.

#### Caffeine

All responding units use caffeine, and about 70% of them prefer a brand caffeine. Doxapram is used in 4.5% of centers in Italy and 14.8% in Europe (*p* = 0.014). The median (IQR) bolus dose of caffeine in Italy and in Europe is 20 mg/kg (20-20), while the highest maintenance dose in Italy and in Europe is 10 (5-10) mg/kg. In Italy and in Europe, prophylactic caffeine in infants with a birth weight (BW) < 1250 g is given in a similar way: in the DR or within 2 h of life in about 74% of units; within 12 h of life in about 10%; within 24 h of life in about 9%; and within 3 days of life in slightly more than 1%. Prophylactic caffeine is not given in about 3% of the participating centers. Finally, caffeine or other methylxanthines are given to intubated infants in about 90% of Italian and European units.

#### Steroids

Postnatal steroids are given from the second week of life in about 30% of the Italian and European units, while 53.3% vs. 39.6% of them start giving postnatal steroids from the third week of life. Postnatal steroids are not used in 5.6% of units in Italy and in 13.1% of units in Europe (*p* = 0.018). Accordingly, 10.2% of academic European NICUs do not use postnatal steroids, compared to 24.1% of non-academic ones (*p* = 0.053). Steroids are given to facilitate extubation in slightly more than 70% of Italian and European units and to infants at high risk of BPD, regardless of ventilation mode, in about 50% of them. In about 50% of Italian and European responding units the favourite strategy is to give low dose dexamethasone. Standard dose dexamethasone is given in 25.9% of Italian vs. 19.3% of European NICUs, while hydrocortisone in 12.9% vs. 27.0%, respectively (*p* = 0.010). Budesonide + surfactant is used in only 2.4% of the responding centers, in Italy and in Europe. The number of allowed cycles of postnatal steroids is similar between Italian and European NICUs. In Italy 37.6% of NICUs administer maximum 1 cycle and 61.2% 2–3 cycles, while in Europe the percentage is 47.1% and 51.2%, respectively (*p* = 0.283). Inhaled steroids are used in 42.3% of Italian vs. 65.4% of European NICUs (*p* < 0.001).

### The mechanical ventilators available in NICU

**Fig. 7:**
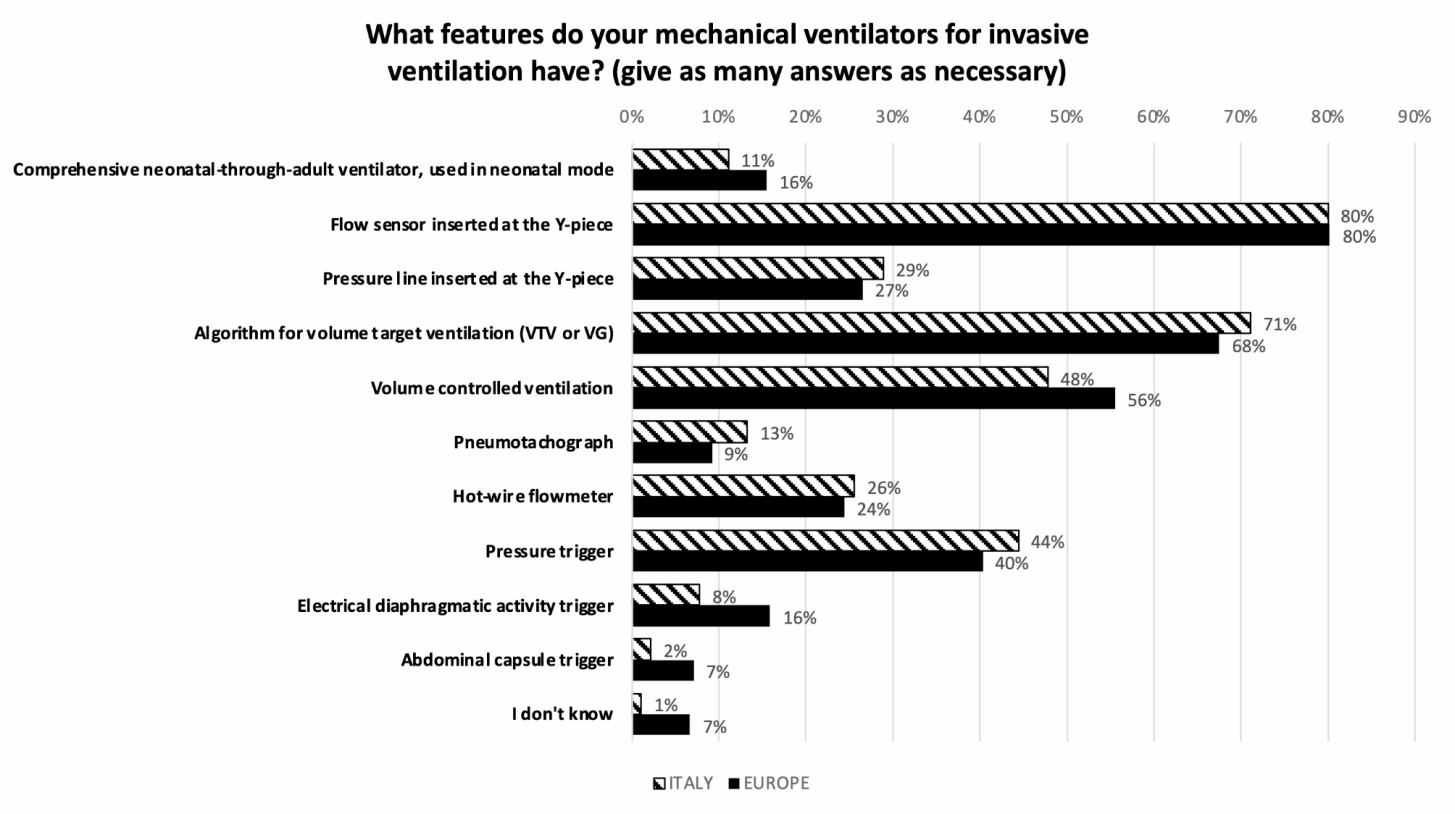
Features of mechanical ventilators for invasive ventilation available in NICU. VTV = Volume Targeted Ventilation; VG = Volume Guarantee. Numbers express the percentage of responding centers

**Fig. 8:**
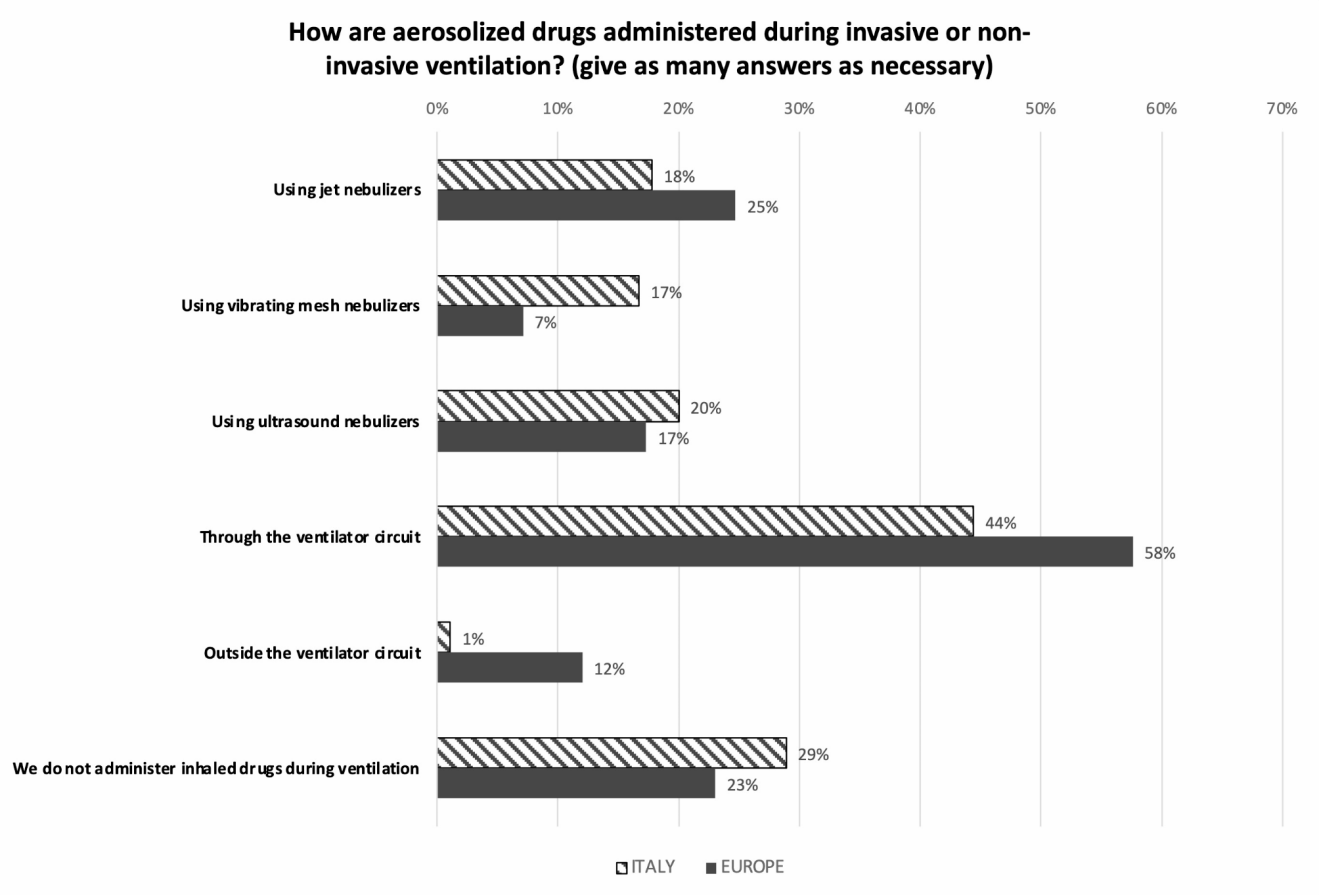
Ways of administering respiratory drugs during invasive and non-invasive ventilation. Numbers express the percentage of responding centers

Figure 7 describes the features of the mechanical ventilators for invasive ventilation available in Italian and European units. Regarding HFOV, in 62.2% of Italian NICUs are available ventilators with active exhalation generated by a piston, in 34.4% ventilators with active exhalation generated by a Venturi effect, and in 23.3% ventilators with active exhalation generated by another method. Only 8.9% NICUs responded to this question “I don’t know”. In Europe, percentages change to 36.4%, 21.9%, and 13.1%, respectively. 43% of European units responded to this question “I don’t know”. Administration modes for aerosolized drugs during invasive or non-invasive ventilation in Italian and European units are indicated in Fig. 8. The gas conditioning mode most used in NICUs for invasive and non-invasive ventilation is the ‘automatic’ mode, indicated by about 75% of the responding centers, while the ‘set up’ mode, with manually set temperatures, is used in the remaining ones. Finally, a standardized protocol for the maintenance of respiratory devices is available in 68.9% of the Italian and in 74.6% of the European centers.

## Discussion

This study provides large scale, up to date results of the current strategies of respiratory care in Italy compared to Europe. Main variations may be partly explained by the paucity of evidence-based data, while the observed differences point to the possibility of implementing “better practices” with the aim of improving neonatal outcomes.

Regarding general information, the survey showed significant differences mostly related to the number of responding centers which were academic hospital in Italy and in Europe, and to the NICUs admission rates of VLBW infants per year. The lower number of Italian responding academic centers compared to the European ones could have represented a bias, as it is expected that introduction in clinical practice and dissemination of the most recent evidence should be more consistent in academic than in non-academic hospitals. According to our survey, academic centers, as expected, tended to be larger on average, as indicated by the higher number of intensive care beds and the greater number of VLBW infants admitted, both in Italy and in Europe. These centers also reported a higher incidence of severe BPD. This association is likely due to the connection between academic neonatology units and academic obstetric services, which typically manage a population of women with more severe pregnancy complications. As a result, these centers care for a case mix characterized by a higher proportion of preterm and critically ill infants. Nevertheless, no relevant differences were observed in the strategies and technologies used for respiratory care when comparing Italian academic and non-academic NICUs, whereas our survey highlighted some significant differences between academic and non-academic European NICUs. A previous survey conducted among Italian birth centers reported similar results [16]. This evidence mostly relies on the fact that the Italian Society of Neonatology (SIN) and its Pulmonology Board heavily invested in organizing educational training courses on neonatal respiratory care. Among the most recent, nationally accredited courses named “Techniques and Technologies for Neonatal Respiratory Care” (Tecniche e Tecnologie per l’Assistenza Respiratoria Neonatale– TTAReN)”, created in collaboration with the bioengineers of the Department of Electronics Information and Bioengineering (DEIB) of Politecnico di Milano (Milan, Italy) to promote adequate medical and technological knowledge on this topic, are available for practitioners and instructors since 2018.

On the other side, the different very low birth weight (VLBW) infants’ admission rates per year reflect different territorial and structural organizations. In Italy, regionalization of perinatal care is an unresolved issue. Actually, more than 100 level II centers are distributed across the Country, serving less than 400.000 births per year. Nevertheless, although regionalized perinatal care seems to be a crucial strategy to improve the survival of VLBW and preterm births, heterogeneous quality of the studies on this topic limits valid conclusions in terms of effectiveness of perinatal regionalisation programmes [17]. In this field, a recent trend towards deregionalization in many US states has been observed, including an increase of the proportion of VLBW infants born outside of Level III centers, as well as a proliferation of smaller neonatal units within the same regions [18]. Reasons for this controversy are related with the potential difficulties resulting from a reduced number of hospitals providing neonatal care, like the need for in-utero transport, the obstacles linked to some peculiar geographical conditions, and the parental sensitivity and perceived discomfort of being away from home for a long time [19].

Moving to the modes, devices and strategies employed in the DR, one of the most relevant differences is related to the lowest GA at which full resuscitation is initiated, being this from 24 weeks on in 21% of the European centers, while in almost all the Italian birth centers the limit is set at 22–23 weeks. The reasons for these differences can be attributed to organizational, cultural, religious, demographic, and economic factors, whose impact remains evident also when considering both EU and non-EU countries. It is evident that neonatologists deal with extremely complex situations when deciding on whether to start, continue or interrupt vital support in infants born at the limit of viability. Nevertheless, currently improving trends in survival and developmental outcomes of extremely preterm infants raise ethical questions, not only for physicians but also for parents and families [20]. Moreover, the inconsistency in treatment practices for infants born between 22 and 24 weeks of GA may account for inter-hospital variation in survival rates, with and without impairment [21]. All these considerations make ethics in neonatology a delicate field that deserves further research.

The most popular device used for respiratory stabilization of preterm infants in the DR is the T-piece ventilator. However, a recently published trial suggests that a novel device, that utilizes the jet technology to provide CPAP/positive pressure ventilation (PPV), may further decrease the work of breathing (WOB) of DR ventilated infants and improve outcomes [15] and for this reason it probably deserves more attention by neonatologists. Since 1994, the SIN and its Task Force on Neonatal Resuscitation adopted the guidelines drawn up by the American Academy of Pediatrics (AAP)/American Heart Association (AHA). This choice reflects the different orientation in setting the initial FiO_2_ to assist infants with GA < 28 weeks, being the European Resuscitation Council guidelines more widespread among the rest of Europe. Surfactant given through the LISA technique is more largely used in the DRs of the European centers compared to Italy. The difference may rely on the observation that the LISA approach, considered as a component of a complex bundle of care supporting premature infants to adapt to extrauterine life through a minimal handling approach, was primarily developed in Germany and western Europe, and only secondarily adopted in Italy. Finally, on surfactant administration modes in the DR, only few participating centers follow the 2022 European Guidelines, that suggest the laryngeal mask as a mean to administer surfactant in infants weighing > 1000 g at birth [22].

In the NICU, HFOV is used as first choice to treat RDS in significantly more Italian centers, and the number even increases when treating high risk preterm infants. Although the scientific literature does not support a clear advantage of elective HFOV compared to elective CMV in treating neonatal RDS [23], HFOV has gained more popularity in Italy than in other European countries. This preference may be partly attributed to the excellent expertise developed in some reference teaching centers in Italy. Notably, the “open lung concept,” which involves a lung-recruitment maneuver to optimize MAP during HFOV, and the volume-targeted HFOV are significantly more frequently performed in Italian NICUs compared to European ones. Additional factors may include the role of scientific societies in organizing training courses and the influence of companies promoting this ventilation method. Similarly, a lung-recruitment procedure to adjust PEEP during CMV, is more often performed among Italian NICUs. While the “open lung concept” is a well-defined strategy aiming at optimal lung recruitment during HFOV [24], studies on PEEP optimization during CMV are still lacking, despite PEEP optimization is one of the most relevant aspects of the “lung-protective ventilation”. Lung-protective ventilation occurs when physiological tidal volumes are uniformly distributed in a homogeneously aerated lung. Indeed, in case of persistent atelectasis or fluid-filled areas, applying the same physiological tidal volumes may result in overinflation of the already opened alveolar units, causing volutrauma and biotrauma. At the same time, these pathologic conditions create “stress raisers” located at the interface between closed and open lung units, where lung injury is augmented by cyclic opening and closing of tidal ventilation [25]. In this view, Castoldi and colleagues proposed a lung recruiting manoeuvre to be applied during SIPPV + VG ventilation, based on FiO_2_ changes [26]. However, a recent Cochrane review concluded that the evidence to guide PEEP level selection for preterm infants on CMV for RDS or BPD continues to be insufficient [27]. More recently, LUS has been proposed as a tool for this purpose [28] while the electrical impedance tomography (EIT) [29] and the respiratory oscillometry [30] are promising technologies to be implemented bedside. Regardless of the geographical area, when weaning infants from MV, the first-choice strategies mainly include modes in which all infant’s spontaneous breaths are supported, being this the most suitable and effective way to support preterm infants during transition towards autonomous breathing. Closed loop automated oxygen control, adopted by about 30% of the responding units, has been shown in short term trials including preterm and low birth weight infants to improve target saturation achievement [31]. However, further investigations are needed to address whether long-term outcomes will be improved with their use. According to our data, NCPAP remains the “gold standard” of NIV. Among pressure generators, several studies indicate that jet generators, like the Infant Flow driver or the Benveniste device, may better support the infant’ spontaneous breathing during both inspiration and expiration, thus reducing WOB compared to bubble CPAP or ventilator-derived CPAP [8]. Although available, these devices are not used as first-choice in many NICUs, probably reflecting the lack of robust evidence in this field. Considering other NIV modes, NIPPV is slightly more used in Italian than in European NICUs, although the difference is not statistically significant. This may represent a sort of “cultural heritage” for some Italian NICUs, as Italy has a long tradition on NIPPV being the first paper on this mode published by Italian authors [32]. Emerging NIV techniques, like NIV-NAVA and NHFOV, are less common, probably reflecting the lack of evidence-based literature in these fields [33, 34]. Moving to interfaces, short binasal prongs and nasal masks, which impose a low resistive WOB are the most widespread. In about 40% of the units DILCs are also available. Despite more resistive, these interfaces are often preferred by parents and nurses as they ensure optimal comfort to the baby and provide a better view of the patient, favouring parents-child bonding. Finally on ventilation modes, the lack of written protocols on how to move from non-invasive to invasive ventilation and vice-versa represents a better-practice to implement in both Italian and Europeans NICUs. As our survey reports, considerable practice style variation exists among the participating hospitals and the lack of consistently high standard of optimal ventilation, most commonly for the acute and weaning phase, may deprive some infants of the benefits of state-of-the-art care. More research is needed to better understand the role of written procedures in reducing unnecessary variations in practice and improving short- and long-term outcomes [35].

Several studies indicate that the ultrasound-guided surfactant replacement improves its timeliness, allowing preterm infants to be treated in the early phase of neonatal RDS, thus improving the course of the disease [36]. The use of LUS in neonatology owes its popularity to Italian authors [37]; however, this tool needs broader implementation in Italian and European NICUs. Similarly to the DR management, surfactant given through the LISA technique is more largely used in the European NICUs compared to the Italian ones. Possible reasons for this disparity have been discussed above. Analgesic/sedative strategies to protect preterm infants during LISA are less used in Europe than in Italy, reflecting the uncertain position of the neonatologists in this regard, as the technique does not spare the infant from the discomfort associated with the visualization of the glottis but, on the other hands, sedation may depress the infant’s spontaneous breathing, especially at lower GAs, reducing the chances of a successful procedure [38]. Continuing on strategies, 80% of the Italian centers perform a lung recruiting manoeuvre before administering surfactant compared to slightly more than half of the European NICUs, despite preclinical and clinical studies show that lung recruitment before surfactant administration may improve gas exchange and lung function as a consequence of more homogeneous surfactant distribution [39]. Similarly, when administering surfactant through an endotracheal tube in mechanically ventilated infants, a closed-circuit should be used, as lung de-recruitment during disconnection from the ventilation circuit may interfere with surfactant distribution and its efficacy.

Supporting spontaneous breathing with early caffeine (i.e. within the first 2 h of life) is the preferred strategy by more than 70% of the participating centers, and literature data support this choice. Dekker et al. observed that DR caffeine significantly increases tidal volumes and decreases the need for oxygen therapy [40] while, more recently, Dani et al. performed a feasibility study to assess whether caffeine administered orally or through the umbilical vein in the DR can reduce the risk of MV in very preterm infants [41]. Finally, Katheria et al. reported that caffeine administration < 2 h from birth decreases the need for MV and results in an overall hemodynamic improvement of treated patients [42]. Among other respiratory stimulants, doxapram is rarely given in cases of apnea refractory to the methylxanthine treatment. Indeed, a recent Cochrane review concludes that more studies are needed to clarify the benefits and harms of doxapram therapy, addressing concerns about long-term outcomes [43].

According to our data, about half of the responding units consider giving postnatal steroids from the third week of life, even if systemic steroids given from the 7th day of life seems not to increase the incidence of long-term adverse outcomes [44]. Therefore, the opportunity to consider their administration starting from the second week of life may facilitate earlier extubation and be more effective in interrupting or slowing down the trajectory of the evolving BPD. Among alternative routes of administration, budesonide conveyed by therapeutic surfactant appears to be extremely promising, although seldom used by participating units. A meta-analysis published in 2022, which included 12 studies, confirmed that the combination of surfactant + budesonide reduces the incidence of BPD, death or BPD and mortality, and follow-up at 36 months of corrected age of the treated infants showed no negative effect on neuromotor and cognitive development [45]. Two large RCTs are currently underway and will provide information on the long-term outcomes associated with this strategy [44].

Finally on the mechanical ventilators, Italian centers seem to be more aware of the technological features of the machines in use in their NICUs, probably reflecting the effect of the network, created also by the above mentioned TTAReN courses, between neonatologist and bioengineers with the aim of improving knowledge on the operating modes of the mechanical ventilators available in NICUs and thus optimizing their use.

Our research highlights both strengths and weaknesses. One of the strengths is the structured questionnaire, which was crafted by a team of experts in the field. Moreover, the study explored various facets of neonatal respiratory management and benefits from a broadly distributed and representative sample. On the other hand, the limitations include low response rates in certain regions, which might introduce selection bias, and the fact that most of the information was provided by neonatal ward directors. This, along with the absence of written protocols or procedures for the areas in question, could lead to a biased perspective. Nevertheless, by sending the survey to the heads of units, we were confident that the leadership of medical directors significantly influences the policies adopted by their units.

## Conclusions

In summary, we conclude that the respiratory support strategies among the surveyed Italian and European NICUs are quite dissimilar in some areas, particularly where high-quality evidence is lacking. The most relevant differences are related to invasive mechanical ventilation strategies, surfactant administration techniques, and technical knowledge about the devices available for neonatal respiratory support. Despite these differences, the incidence of severe BPD is similar across Europe. The next phase of our work will involve providing the participating National Societies with detailed information on RDS management specific to their countries, enabling the creation of an accurate, country-specific overview of neonatal RDS care. This data will help stakeholders to compare national data with overall trends, understand variations in clinical practices, conduct comparative evaluations of outcomes, identify areas for improvement, and develop a European collaborative platform for staying current. Further research is needed in areas such as respiratory care strategies and monitoring at birth, lung recruitment manoeuvres, non-invasive surfactant administration techniques, and simple, reliable synchronization methods for NIV, as these may significantly impact care.

## Electronic supplementary material

Below is the link to the electronic supplementary material.

Supplementary Material 1

Supplementary Material 2

## Data Availability

The datasets used and/or analysed during the current study are available from the corresponding author on reasonable request.

## Abbreviations

AAP: American Academy of Pediatrics
AHA: American Heart Association
ARDS: Acute Respiratory Distress Syndrome
BiPAP: Bilevel Positive Airway Pressure
BPD: Bronchopulmonary Dysplasia
BW: Birth Weight
CMV: Conventional Mechanical Ventilation
DEIB: Department of Electronics Information and Bioengineering
DILC: Double-Inspiratory Loop Cannulas
DR: Delivery Room
ECMO: Extracorporeal Membrane Oxygenation
EIT: Electrical Impedance Tomography
GA: Gestational Age
HFOV: High Frequency Oscillatory Ventilation
INRECSURE: INtubation RECruitment of the lung -SURfactant– Extubation
INSURE: INtubation-SURfactant-Extubation
IQR: Interquartile Range
IVH: Intraventricular Haemorrhage
LISA: Less Invasive Surfactant Administration
LUS: Lung Ultrasound
MAP: Mean Airway Pressure
MIST: Minimally Invasive Surfactant Therapy
MV: Mechanical ventilation
NAVA: Neurally Adjusted Ventilatory Assist
NCPAP: Nasal Continuous Positive Airway Pressure
NDI: Neurodevelopmental Impairment
NHFOV: Nasal High Frequency Oscillatory Ventilation
nHFT: Nasal High Flow Therapy
NICU: Neonatal Intensive Care Unit
NIMV: Nasal Intermittent Mandatory Ventilation
NIPPV: Nasal Intermittent Positive Pressure Ventilation
NIV-NAVA: Non-Invasive Ventilation-Neurally Adjusted Ventilatory Assist
NIV: Non-Invasive Ventilation
NO: Nitric Oxide
PAV: Proportional Assist Ventilation
PEEP: Positive End Expiratory Pressure
PPROM: Preterm Premature Rupture of Membranes
PPV: Positive Pressure Ventilation
PSV: Pressure Support Ventilation
RCT: Randomized Controlled Trial
RDS: Respiratory Distress Syndrome
RFM: Respiratory Function Monitoring
SIN: Società Italiana di Neonatologia (Italian Society of Neonatology)
SIPPV: Synchronized intermittent positive pressure ventilation
SNIMV: Synchronized Nasal Intermittent Mandatory Ventilation
SNIPPV: Synchronized Nasal Intermittent Positive Pressure Ventilation
Ti: Inspiratory time
TTAReN: Tecniche e Tecnologie per l’Assistenza Respiratoria Neonatale
UENPS: Union of European Neonatal and Perinatal Societies
VG: Volume Guarantee
VLBW: Very Low Birth Weight
VTV: Volume-Targeted Ventilation
WOB: Work of Breathing
