# Supplementary material for: Comparing Italian versus European strategies and technologies for respiratory care in NICU: results of a survey of the Union of European Neonatal and Perinatal Societies (UENPS) and the Italian Society of Neonatology (SIN)
Source: Ital J Pediatr. 2025 Mar 26;51:100. doi: 10.1186/s13052-025-01936-6 (PMC11948922; doi:10.1186/s13052-025-01936-6)
Supplement: Supplementary file 2 — Supplementary Material 2 [file 13052_2025_1936_MOESM2_ESM.pdf]

PARTICIPATING NICUS

UNIVERSITY HOSPITAL FOR OBSTETRICS AND GYNECOLOGY "KOÇO GLIOZHENI", TIRANA, ALBANIA

SLAVMED MC, YEREVAN, ARMENIA

MEDICAL UNIVERSITY OF INNSBRUCK, INNSBRUCK, AUSTRIA|LANDESKRANKENHAUS, SALZBURG, AUSTRIA

GOMEL REGIONAL CLINICAL HOSPITAL, GOMEL, BELARUS|CLINICAL MATERNITY HOSPITAL OF MINSK REGION, MINSK, BELARUS|PERINATAL CENTER GRODNO, GRODNO, BELARUS|NATIONAL RESEARCH AND PRACTICAL CENTER MOTHER AND CHILD, MINSK, BELARUS

ZIEKENHUIS OOST-LIMBURG, GENK, BELGIUM|CHU MARIE CURIE CHARLEROI, LODELINSART, BELGIUM|CLINIQUE CHCMONTLEGIA, LIÈGE, BELGIUM|UNIVERSITEITS ZIEKENHUIS ANTWERPEN, ANTWERPEN, BELGIUM|UZ BRUSSEL - VRIJE UNIVERSITEIT BRUSSEL, BRUSSELS, BELGIUM|ZNA MIDDELHEIM, ANTWERPEN, BELGIUM|CHU-CHR LIÈGE, LIÈGE, BELGIUM|HOPITAL ERASME, BRUXELLES, BELGIUM|AZ SINT-JAN BRUGGE-OOSTENDE AV, BRUGGE, BELGIUM|UZ LEUVEN, LEUVEN, BELGIUM|HÔPITAL UNIVERSITAIRE DES ENFANTS REINE FABIOLA, BRUSSELS, BELGIUM|SAINT-LUC UNIVERSITY HOSPITAL, UCLOUVAIN, BRUSSELS, BELGIUM

PAEDIATRIC HOSPITAL, SARAJEVO, BOSNIA AND HERZEGOVINA|CLINIC OF CHILDRENS'S DISEASES BANJA LUKA, BANJA LUKA, BOSNIA AND HERZEGOVINA|UNIVERSITY HOSPITAL MOSTAR, MOSTAR, BOSNIA AND HERZEGOVINA|PEDIATRICS CLINIC, UNIVERSITY CLINICAL CENTER TUZLA, TUZLA, BOSNIA AND HERZEGOVINA

UMHAT ST GEORGE, PLOVDIV, BULGARIA|UNIVERSITY HOSPITAL "DR. GEORGI STRANSKI, PLEVEN, BULGARIA|UNIVERSITY PEDIATRIC HOSPITAL, SOFIA, BULGARIA|UNIVERSITY HOSPITAL, STARA ZAGORA, BULGARIA|UNIVERSITY HOSPITAL OF OBSTETRICS AND GYNECOLOGY "MAICHIN DOM", SOFIA, BULGARIA|UNIVERSITY HOSPITAL, PLEVEN, BULGARIA|SPECIALISED OB/ GYN HOSPITAL "SHEJNOVO", SOFIA, BULGARIA

UNIVERSITY HOSPITAL CENTER SESTRE MILOSRDNICE, ZAGREB, ZAGREB, CROATIA|CLIN.CENT. RIJEKA, RIJEKA, CROATIA|UNIVERSITY HOSPITAL OSIJEK, OSIJEK, CROATIA|CLINICAL HOSPITAL CENTER ZAGREB, ZAGREB, CROATIA|CLINICAL HOSPITAL "HOLY SPIRIT", ZAGREB, CROATIA|UNIVERSITY HOSPITAL CENTRE ZAGREB, ZAGREB, CROATIA|CLINICAL HOSPITAL CENTRE SPLIT, ŠIBENIK, CROATIA

UNIVERSITY HOSPITAL MOTOL, PRAHA, CZECH REPUBLIC |KRAJSKA ZDRAVOTNI, MASARYK HOSPITAL, USTI NAD LABEM, CZECH REPUBLIC |GENERAL UNIVERSITY HOSPITAL, PRAGUE, CZECH REPUBLIC |UNIVERSITY HOSPITAL BRNO, BRNO, CZECH REPUBLIC |TOMAS BATA HOSPITAL, ZLÍN, CZECH REPUBLIC |UNIVERZITY HOSPITAL, OSTRAVA, CZECH REPUBLIC |UNIVERSITY HOSPITAL OLOMOUC, OLOMOUC, CZECH REPUBLIC |HOSPITAL MOST, MOST 1, CZECH REPUBLIC |HOŘOVICE, HIŘOVICE, CZECH REPUBLIC |UNIVERSITY HOSPITAL HRADEC KRALOVE, HRADEC KRALOVE, CZECH REPUBLIC

RIGSHOSPITALET, KØBENHAVN, DANMARK|LILLEBELT HOSPITAL, KOLDING, DENMARK|AALBORG UNIVERSITY HOSPITAL, AALBORG, DENMARK|REGIONHOSPITAL NORDJYLLAND, HJØRRING, DENMARK|NYKOEBING F, NYKOEBING F, DENMARK

TALLINN CHILDREN`S HOSPITAL, TALLINN, ESTONIA|TARTU UNIVERSITY HOSPITAL, TARTU, ESTONIA|EAST TALLIN CENTRAL HOSPITAL, TALLINN, ESTONIA

HUS, HELSINKI UNIVERSITY HOSPITAL, HELSINKI, FINLAND|TURKU UNIVERSITY HOSPITAL, TURKU, FINLAND|OULU UNIV HOSP, OULU, FINLAND|KUOPIO UNIVERSITY HOSPITAL, KUOPIO, FINLAND

COCHIN-PORT ROYAL, PARIS, FRANCE|CENTRE HOSPITALIER INTERCOMMUNAL DE CRÉTEIL, CRÉTEIL, FRANCE|CHU BORDEAUX, BORDEAUX, FRANCE|CH DE MANTES LA JOLIE, MANTES LA JOLIE, FRANCE|LILLE UNIVERSITY HOSPITAL, LILLE, FRANCE|CENTRE HOSPITALIER D'AUXERRE, AUXERRE, FRANCE|CALAIS, CALAIS, FRANCE|MFME, FORT DE FRANCE, FRANCE|CHU ESTAING, CLERMONT-FERRAND, FRANCE|GHRMSA, MULHOUSE, FRANCE|CENTRE HOSPITALIER DE SAINT-DENIS, SAINT-DENIS, FRANCE|CHI POISSY SAINT-GERMAIN-EN-LAYE, POISSY, FRANCE|CHU CARÉMEAU, NÎMES, FRANCE|JEANNE DE FLANDRE, LILLE, FRANCE|HÔPITAL FEMME MÈRE ENFANT, LYON, FRANCE|HÔPITAL INTERCOMMUNAL DE CRETEIL, CRETEIL, FRANCE|TROYES, TROYES, FRANCE| FELIX GUYON, BELLEPIERRE, LA REUNION, FRANCE| CHPF, PAMATAI, POLYNÉSIE FRANÇAISE

UNIVERSITY MEDICINE GÖTTINGEN, GÖTTINGEN, GERMANY|UNIVERSITY HOSPITAL, DRESDEN, GERMANY|UNIVERSITY HOSPITAL FREIBURG, FREIBURG, GERMANY|MARIEN-HOSPITAL WESEL, WESEL, GERMANY|CHILDRENS HOSPITAL

PASSAU, PASSAU, GERMANY|HAUNER CHILDRENS HOSPITAL, MUNICH, GERMANY|UNIVERSITY OF WÜRZBURG, WURZBURG, GERMANY |UNIVERSITÄTSKLINIK BONN, BONN, GERMANY |UNIVERSITY, FREIBURG, GERMANY

MITERA MATERNITY HOSPITAL, ATHENS, GREECE|UNIVERSITY GENERAL HOSPITAL ATTIKON, ATHENS, GREECE|LETO MATERNITY HOSPITAL, ATHENS, GREECE|PAPAGEORGIOU GENERAL HOSPITAL, THESSALONIKI, GREECE|UNIVERSITY GENERAL HOSPITAL OF ALEXANDROUPOLIS, ALEXANDROUPOLI, GREECE|ATTIKON UNIVERSITY HOSPITAL, ATHENS, GREECE|UNIVERSITY GENERAL HOSPITAL OF PATRAS, PATRAS, GREECE|GENERAL HOSPITAL OF PATRAS "AGIOS ANDREAS", PATRAS, GREECE| ALEXANDRA GENERAL HOSPITAL, ATHENS, GREECE|REA MATERNITY HOSPITAL S.A., FALIRO, GREECE|AGIA SOPHIA CHILDRENS HOSPITAL OF ATHENS, ATHENS, GREECE|MAT. HOSP. ELENA VENIZELOU, ATHENS, GREECE|UNIVERSITY HOSPITAL IOANNINA, IOANNINA, GREECE|UNIVERSITY HOSPITAL FO HERAKLION, HERAKLION, GREECE|IPPOKRATIO GENERAL HOSPITAL, THESSALONIKI, GREECE|HIPPOKRATION GENERAL HOPITAL, THESSALONIKI, GREECE|IASO MATERNITY HOSPITAL, ATHENS, GREECE

PETZ ALADÁR UNIVERSITY TEACHING HOSPITAL, GYŐR, HUNGARY|MARKUSOVSZKY HOSPITAL, SZOMBATHEHELY, HUNGARY|HEIM PAL CHILDEN HOSPITAL, BUDAPEST, HUNGARY|BAROSS STREET DEPARTMENT OF OBSTETRICS AND GYNECOLOGY, BUDAPEST, HUNGARY|SOPRONI GYOGYKOZPONT, SOPRON, HUNGARY|SEMMELWEIS UNIVERSITY, BUDAPEST, HUNGARY|UNIVERSITY OF PÉCS, PÉCS, HUNGARY|COUNTY HOSPITAL, NYÍREGYHAZA, HUNGARY|CLINICAL CENTRE UNIVERSITY OF DEBRECEN, DEBRECEN, HUNGARY|BÁCS-KISKUN COUNTY HOSPITAL, KECSKEMÉT, HUNGARY|CSOLNOKY FERENC HOSPITAL, VESZPREM, HUNGARY|PÉTERFY SÁNDOR U. KÓRHÁZ, BUDAPEST, HUNGARY|FMSZGYEOK, SZÉKESFEHÉRVÁR, HUNGARY |BMKK PÁNDY KÁLMÁN HOSPITAL, GYULA, HUNGARY

ZIV MEDICAL CENTER, SAFED, ISRAEL|SCHNEIDER CHILDREN'S MEDICAL CENTER, PETACH TIKVA, ISRAEL|BNAI ZION, HAIFA, ISRAEL|FRENCH HOSPITAL- ISRAEL, NAZARETH, ISRAEL|SOROKA MEDICAL CENTER, BEER SHEVA, ISRAEL|MAYANEI HAYESHUA, BNEI BRAK, ISRAEL|SHAMIR MEDICAL CENTER, ZERIFFIN, ISRAEL |HADASSAH, JERUSALEM, ISRAEL

VILLA DEI FIORI, ACERRA, ITALY|SPIRITO SANTO, PESCARA, ITALY|SAN LEONARDO, CASTELLAMMARE DI STABIA, ITALY|VERSILIA, VIAREGGIO, ITALY|UMBERTO I, ENNA, ITALY|CLINICA MALZONI-VILLA DEI PLATANI, AVELLINO, ITALY|PO UMBERTO 1°, SIRACUSA, ITALY|AUSL-IRCCS REGGIO EMILIA, REGGIO EMILIA, ITALY|SANNFRANCESCO, NUORO, ITALY|AOUPOLICNICO G RODOLICO SAN MARCO PRESIDIO RODOLICO, CATANIA, ITALY|BUON CONSIGLIO FATEBENEFRATELLI, NAPOLI, ITALY|STANTO STEFANO PRATO, PRATO, ITALY|FATEBENEFRATELLI DI BENVENTO, BENEVENTO, ITALY|"A. CARDARELLI", CAMPOBASSO, ITALY|BAMBINO GESÙ CHILDREN'S HOSPITAL, ROMA, ITALY|GRANDE OSPEDALE METROPOLITANO, REGGIO CALABRIA, ITALY|SAN SALVATORE, L'AQUILA, ITALY|OSPEDALE SANTA CHIARA, TRENTO, ITALY|P.O. "A.PERRINO" - ASL BRINDISI - ITALY, BRINDISI, ITALY|PINETA GRANDE, CASTEL VOLTURNO, ITALY|AZ. OSPEDALIERA S. MARIA TERNI, TERNI, ITALY|SAN GIUSEPPE MOSCATI, AVELLINO, ITALY|CASA SOLLIEVO DELLA SOFFERENZA SAN GIOVANNI ROTONDO, MANFREDONIA, ITALY|BARONE ROMEO, PATTI, ITALY|SAN PIETRO FATEBENEFRATELLI, ROMA, ITALY|SAN GERARDO - MONZA, MONZA, ITALY|BOLOGNINI, SERIATE, ITALY|AZIENDA OSPEDALIERA UNIVERSITÀ DI PADOVA, PADOVA, ITALY|ARNAS GARIBALDI, CATANIA, ITALY|SANTA CHIARA HOSPITAL - PISA, CASCINA, ITALY|CARLO POMA, MANTOVA, ITALY|INGRASSIA, PALERMO, ITALY|ASST PAPA GIOVANNI XXIII, BERGAMO, ITALY|FONDAZIONE IRCCS SAN MATTEO, PAVIA, ITALY|SAN CAMILLO, ROMA, ITALY|CA' FONCELLO REGIONAL HOSPITAL, TREVISO, ITALY|A.O. DEI COLLI OSPEDALE MONALDI, NAPOLI, ITALY|"V.BUZZI" OSPEDALE DEI BAMBINI, MILANO, ITALY|AOU SALERNO, SALERNO, ITALY|SAN DONATO AREZZO, AREZZO, ITALY|AOU SASSARI, SASSARI, ITALY|DONNA E BAMBINO, OSPEDALE CIVILE MAGGIORE, VERONA, ITALY|UNIVERSITY CHILDREN HOSPITAL OF PARMA, PARMA, ITALY|DEL PONTE, VARESE, ITALY|POLICLINICO DUILIO CASULA, MONSERRATO, ITALY|POLICLINICO, BARI, ITALY|AOU G. MARTINO UNIVERSITY OF MESSINA, MESSINA, ITALY|SANTOBONO, NAPLES, ITALY|INFERMI RIMINI, RIMINI, ITALY|PO S. ABATE TRAPANI, TRAPANI, ITALY|ARNAS CIVICO, PALERMO, ITALY|SAN VINCENZO DI TAORMINA, TAORMINA, ITALY|AORN A. CARDARELLI, NAPLES, ITALY|MACEDONIO MELLONI ASST FBF-SACCO, MILAN, ITALY|SANT'EUGENIO, ROME, ITALY|ST ORSOLA GENERAL HOSPITAL - UNIVERSITY OF BOLOGNA, BOLOGNA, ITALY|AOU VANVITELLI, NAPOLI, ITALY|ASST SPEDALI CIVILI DI BRESCIA, BRESCIA, ITALY|AORN SAN PIO - BENEVENTO ITALY, BENEVENTO, ITALY|FEDERICO II, NAPLES, ITALY|OSPEDALE MAGGIORE, BOLOGNA, ITALY|AZIENDA OSPEDALIERA SAN GIOVANNI ADDOLORATA, ROMA, ITALY|SANT'ANNA, SAN FERMO DELLA BATTAGLIA, ITALY|AZIENDA OSPEDALIERA PERUGIA, PERUGIA, ITALY|ASP KR- OSPEDALE SAN GIOVANNI DI DIO, CROTONE, ITALY|ASST GRANDE OSPEDALE METROPOLITANO NIGUARDA, MILANO, ITALY|FONDAZIONE POLICLINICO UNIVERSITARIO AGOSTINO GEMELLI-IRCCS-UNIVERSITÀ CATTOLICA DEL SACRO CUORE, ROME, ITALY|S. GIOVANNI CALIBITA FBF, ROMA, ITALY|SAN CARLO, POTENZA, ITALY|AZIENDA OSPEDALIERO-UNIVERSITARIA DI FERRARA, FERRARA, ITALY|POLICLINICO CASILINO, ROME, ITALY|POLICLINICO RIUNITI FOGGIA, FOGGIA, ITALY|ANNUNZIATA HOSPITAL, COSENZA, ITALY|SAN GIOVANNI DI DIO, FLORENCE, ITALY|OSPEDALE GENERALE MIULLI, ACQUAVIVA DELLE FONTI, ITALY|IRCCS GASLINI, GENOVA, ITALY|FONDAZIONE IRCCS CA' GRANDA OSPEDALE MAGGIORE POLICLINICO, MILANO, MILANO, ITALY|CAREGGI UNIVERSITY HOSPITAL OF FLORENCE, FLORENCE, ITALY|AOU POLICLINICO UMBERTO I, ROME, ITALY|CANNIZZARO, CARANIA, ITALY|SANT’ANNA AND SAN SABASTIANO, CASERTA, CASERTA,

ITALY|POLICLINICO DI MODENA, MODENA, ITALY|OSPEDALE EVANGELICO BETANIA, NAPOLI, ITALY|DI VENERE ASL BARI, BARI, ITALY|CARD. G. PANICO, TRICASE, ITALY|GIOVANNI PAOLO II, RAGUSA, RAGUSA, ITALY|MANZONI - LECCO, LECCO, ITALY|BOLZANO, BOLZANO, ITALY|SS. NNUNZIATA, TARANTO, ITALY|AOR VILLA SOFIA-CERVELLO, PALERMO, ITALY|VICENZA, SAN BORTOLO HOSPITAL, VICENZA, ITALY|AGRIGENTO, AGRIGENTO, ITALY|BELCOLLE VITERBO, VITERBO, ITALY|POLIAMBULANZA FOUNDATION HOSPITAL, BRESCIA, ITALY|PUGLIESE CIACCIO, CATANZARO, ITALY

PERINATAL CENTR, ALMATY, KAZAKHSTAN|MULTIFUNCTIONAL HOSPITAL, NURSULTAN, KAZAKHSTAN|OPC, TURKISTAN, KAZAKHSTAN|GMB2, NUR-SULTAN, KAZAKHSTAN|SEMEY PERINATAL CENTER, SEMEY, KAZAKHSTAN|SEMEY CITY PERINATAL CENTER, SEMEY, KAZAKHSTAN|MEDICAL UNIVERSITY ASTANA, NUR-SULTAN, KAZAKHSTAN|MULTIDISCIPLINARY CENTER FOR MOTHER AND CHILD, TEMIRTAU, KAZAKHSTAN|PERINATAL CENTER OF QARAGHANDY CITY, QARAGHANDY, KAZAKHSTAN

UNIVERSITY CLINICAL CENTER KOSOVA, PRISHTINA, KOSOVO

P. STRADINS UH, RIGA, LATVIA|RIGA MATERNITY HOSPITAL, RIGA, LATVIA|LIEPĀJA REGIONAL HOSPITAL, LIEPĀJA, LATVIA|RESPUBLIKINĖ ŠIAULIŲ LIGONINĖ, ŠIAULIAI, LATVIA|VILNIUS UNIVERSITY HOSPITAL SANTAROS KLINIKOS, VILNIUS, LATVIA

KLAIPĖDA UNIVERSITY HOSPITAL, KLAIPĖDA, LITHUANIA|CLINICAL HOSPITAL OF LITHUANIAN UNIVERSITY HEALTH SCIENCES, KAUNAS, LITHUANIA|RESPUBLIKINE PANEVĖŽIO LIGONINĖ, PANEVĖŽYS, LITHUANIA

INSTITUTE OF CHILDREN'S DISEASES, PODGORICA, MONTENEGRO

UTRECHT MEDICAL CENTER/WKZ, UTRECHT, NETHERLANDS|LEIDEN UNIVERSITY MEDICAL CENTER, LEIDEN, NETHERLANDS |RADBOUDUMC, NIJMEGEN, NETHERLANDS |AMSTERDAM UMC, EMMA CHILDREN'S HOSPITAL, AMSTERDAM, NETHERLANDS |BEATRIX CHILDRENS HOSPITAL, UMCG, GRONINGEN, NETHERLANDS

UNIVERSITY CHILDREN'S HOSPITAL, SKOPJE, NORTH MACEDONIA|ACIBADEM SISTINA SKOPJE, SKOPJE, NORTH MACEDONIA|UNIVERSITY CLINIC FOR GYN. AND OBSTETRIC, SKOPJE, NORTH MACEDONIA

UNIVERSITY HOSPITAL OF NORTH NORWAY, TROMSØ, NORWAY|DRAMMEN HOSPITAL, DRAMMEN, NORWAY|HAMMERFEST SYKEHUS, HAMMERFEST, NORWAY|AKERSHUS UNIVERSITY HOSPITAL, LØRENSKOG, NORWAY|NORDLAND HOSPITAL, BODOE, NORWAY|FØRDE CENTRAL HOSPITAL, FØRDE, NORWAY|OSLO UNIVERSITY HOSPITAL, OSLO, NORWAY|ÅLESUND HOSPITAL, ÅLESUND, NORWAY|HAUKELAND UNIVERSITY HOSPITAL, BERGEN, NORWAY

MEDICAL UNIVERSITY OF WARSAW, WARSAW, POLAND|UNIVERSITY HOSPITAL NO 2, BYDGOSZCZ, POLAND|CLINICAL PROVINCIAL HOSPITAL, RZESZOW, POLAND|UPPER SILESIAN CHILD'S HEALTH CENTRE, CLINIC HOSPITAL NO6, KATOWICE, POLAND|UNIVERSITY HOSPITAL, KRAKÓW, POLAND|SPSK2 PUM, SZCZECIN, POLAND

CHUA - UNIDADE DE FARO, FARO, PORTUGAL|CENTRO HOSPITALAR DE LEIRIA, LEIRIA, PORTUGAL|CHUCOVA DA BEIRA, COVILHA, PORTUGAL|CUF PORTO, PORTO, PORTUGAL|UNIDADE LOCAL SAÚDE ALTO MINHO, VIANA CASTELO, PORTUGAL|CENTRO HOSPITALAR TONDELA VISEU, VISEU, PORTUGAL|HOSPITAL DO DIVINO ESPIRITO SANTO, PONTA DELGADA, PORTUGAL|ESPÍRITO SANTO, ÉVORA, PORTUGAL|CHULC, LISBOA, PORTUGAL|HOSPITAL PEDRO HISPANO, MATOSINHOS, PORTUGAL|HOSPITAL DE BRAGA, BRAGA, PORTUGAL|BEATRIZ ANGELO, LOURES, PORTUGAL|MATERNIDADE DR. ALFREDO DA COSTA, LISBON, PORTUGAL|GARCIA DE ORTA, ALMADA, PORTUGAL|HOSPITAL DR NELIO MENDONCA, FUNCHAL, PORTUGAL|HOSPITAL SÃO FRANCISCO XAVIER- CENTRO HOSPITALAR DE LISBOA OCIDENTAL, LISBOA, PORTUGAL|CENTRO HOSPITALAR UNIVERSITÁRIO LISBOA CENTRAL, LISBOA, PORTUGAL|HOSPITAL SANTA MARIA - CHULN, LISBOA, PORTUGAL|CENTRO HOSPITALAR UNIVERSITÁRIO DE SÃO JOÃO, PORTO, PORTUGAL|CENTRO HOSPITALAR VILA NOVA DE GAIA / ESPINH, VILA NOVA DE GAIA, PORTUGAL|CENTRO MATERNO INFANTIL DO NORTE, PORTO, PORTUGAL|CENTRO HOSPITALAR UNIVERSITÁRIO DE COIMBRA, COIMBRA, PORTUGAL|VILA FRANCA DE XIRA, VILA FRANCA DE XIRA, PORTUGAL |HOSPITAL DE CASCAIS DR JOSÉ DE ALMEIDA, ALCABIDECHE, PORTUGAL |FERNANDO FONSECA, AMADORA, PORTUGAL

DR I CANTACUZINO HOSPITAL, BUCHAREST, ROMANIA|EMERCENCY CLINICAL HOSPITAL FOR CHILDREN "L.TURCANU", TIMISOARA, ROMANIA|CLINICAL HOSPITAL OF OBSTETRICS-GYNECOLOGY DR. I. A. SBARCEA BRASOV, BRASOV, ROMANIA|"SFANTUL IOAN CEL NOU" EMERGENCY HOSPITAL SUCEAVA, SUCEAVA, ROMANIA|MATERNITY, TIMISOARA, ROMANIA|SPITALUL CLINIC JUDETEAN DE URGENTA CRAIOVA, CRAIOVA, ROMANIA|SCJU CONSTANTA, CONSTANTA, ROMANIA|SCOG PANAIT SIRBU, BUCHAREST, ROMANIA|MUNICIPAL EMERGENCY HOSPITAL - MATERNITY ODOBESCU-TIMISOARA „LOUIS TURCANU" EMERCENCY CHILDREN HOSPITAL – TIMISOARA, TIMISOARA, ROMANIA|MS CURIE CLINICAL EMERGENCY HOSPITAL, BUCHAREST, ROMANIA|INSMC POLIZU, BUCHAREST, ROMANIA|CLINICAL COUNTY HOSPITAL ORADEA, ORADEA, ROMANIA|EMERGENCY COUNTY HOSPITAL, TIRGU MURES, ROMANIA|CUZA-VODA CLINICAL HOSPITAL OF OBSTETRICS AND GYNECOLOGY, IASI, ROMANIA|EMERGENCY COUNTRY HOSPITAL, CLUJ NAPOCA, ROMANIA|CLINICAL COUNTY EMERGENCY HOSPITAL, SIBIU, ROMANIA|SJU BACAU, BACAU, ROMANIA|SCJU

CONSTANTA, CONSTANTA, ROMANIA|SF.PANTELIMON, BUCURESTI, ROMANIA|SP . SF IOAN - MATERNITATEA BUCUR, BUCUREȘTI, ROMANIA|SPITALUL JUDEȚEAN DE URGENTA TÂRGOVIȘTE, TÂRGOVISTE, ROMANIA|CLINICAL HOSPITAL FILANTROPIA, BUCHAREST, ROMANIA

REGIONAL CHILDREN HOSPITAL, EKATERINBURG, RUSSIA

MOTHER AND CHILD HEALTH CARE INSTITUTE OF SEBIA "DR VUKAN CUPIC", BELGRADE, SERBIA|CLINIC FOR GYNECOLOGY AND OBSTETRICS CLINICAL CENTER OF SERBIA, BELGRADE, SERBIA|INSTITUT FOR NEONATOLOGY, BELGRADE, SERBIA|UKC KRAGUJEVAC, KRAGUJEVAC, SERBIA|INSTITUTE OF CHILD AND YOUTH HEALTHCARE OF VOJVODINA, NOVI SAD, SERBIA|UNIVERSITY CHILDREN'S HOSPITAL, BELGRADE, SERBIA

FAKULTNÁ NEMOCNICA, TRNAVA, SLOVAKIA|UNIVERSITY HOSPITAL BRATISLAVA, BRATISLAVA, SLOVAKIA|FACULTY HOSPITAL, NOVE ZAMKY, SLOVAKIA

UNIVERSITY CHILDREN HOSPITAL, LJUBLJANA, SLOVENIA|UKC MARIBOR, MARIBOR, SLOVENIA|UNIVERSITY MEDICAL CENTRE, LJUBLJANA, SLOVENIA

LA PAZ UNIVERSITY HOSPITAL, MADRID, SPAIN|H. SAN PEDRO DE ALCÁNTARA, CÁCERES, SPAIN|HOSPITAL GENERAL UNIVERSITARIO ALICANTE, ALICANTE, SPAIN|H. GENERAL UNIVERSITARIO DE CASTELLON, CASTELLÓN DE LA PLANA, SPAIN|UNIVERSITARIO LA PAZ, MADRID, SPAIN|SAN PEDRO, LOGROÑO, SPAIN|HOSPITAL UNIVERSITARIO DE GETAFE, MADRID, SPAIN|HOSPITAL CLINIC BARCELONA, BARCELONA, SPAIN|PUERTA DEL MAR, SAN FERNANDO, SPAIN|RIO HORTEGA, VALLADOLID, SPAIN|HRU MALAGA, MALAGA, SPAIN|MATERNO-INFANTIL DE LAS PALMAS, LAS PALMAS DE GRAN CANARIA, SPAIN|LA FE HOSPITAL, VALENCIA, SPAIN|HSOPITAL DE MERIDA, MERIDA, SPAIN|HOSPITAL UNIVERSITARIO MIGUEL SERVET, ZARAGOZA, SPAIN|HOSPITAL UNIVERSITARI GERMANS TRIAS I PUJOL, BADALONA, SPAIN|LA FE, VALENCIA, SPAIN|HOSPITAL UNIVERSITARIO DE MÓSTOLES, MÓSTOLES, SPAIN|HOSPITAL CLINIC BARCELONA, BARCELONA, SPAIN|H. UNIVERSITARIO CENTRAL DE ASTURIAS, OVIEDO, SPAIN|HOSPITAL UNIVERSITARIO DE JEREZ, JEREZ DE LA FRONTERA, SPAIN|HRU MALAGA, MALAGA, SPAIN|HOSPITAL UNIVERSITARIO DE NAVARRA, PAMPLONA, SPAIN|SANTA LUCÍA, CARTAGENA, SPAIN|JUAN RAMÓN JIMÉNEZ, HUELVA, SPAIN|TORRECÁRDENAS, ALMERIA, SPAIN|HOSPITAL UNIVERSITARIO MARQUÉS DE VALDECILLA, SANTANDER, SPAIN|HOSPITAL UNIVERSITARI DE TARRAGONA JOAN XXIII, TARRAGONA, SPAIN|HOSPITAL UNIVERSITRI ARNAU DE VILANOVA, LLEIDA, SPAIN|VIRGEN DE LAS NIEVES., GRANADA, SPAIN

UPPSALA UNIVERSITY CHILDREN'S HOSPITAL, UPPSALA, SWEDEN

UNIVERSITY HOSPITAL CENTER LAUSANNE, LAUSANNE, SWITZERLAND|UNIVERSITY HOSPITALS GENEVA, GENEVA, SWITZERLAND|CANTONAL HOSPITAL AARAU, AARAU, SWITZERLAND|HUG, GENEVA, SWITZERLAND

ISTANBUL MEDIPOL UNIVERSITY, ISTANBUL, TURKEY|ALTUNIZADE ACIBADEM, ISTANBUL/USKUDAR, TURKEY|BASKENT UNIVERSITY, ANKARA, TURKEY|HEALTH SCIENCES UNIVERSITY UMRANIYE RESEARCH AND EDUCATION HOSPITAL, ISTANBUL, TURKEY|ADANA CITY TRAINING AND RESEARCH HOSPITAL, ADANA, TURKEY|ULUDAG UNIVERSITY, BURSA, TURKEY|GAZI UNIVERSITY HOSPITAL, ANKARA, TURKEY|KARTAL DR LUTFI KIRDAR CITY HOSPITAL, ISTANBUL, TURKEY|ESKIŞEHIR OSMANGAZI ÜNIVERSITY, ESKIŞEHIR, TURKEY|İSTANBUL MEDENIYET UNIVERSITY HOSPITAL, İSTANBUL, TURKEY|BASAKSEHIR CAM AND SAKURA CITY HOSPITAL, BAŞAKŞEHIR, TURKEY|MALTEPE UNIVERSITY, ISTANBUL, TURKEY|CUKUROVA UNIVERSITY BALCALI HOSPITAL, ADANA, TURKEY|İSTANBUL RESEARCH AND TRAINING HOSPITALL, İSTANBUL, TURKEY|UNIVERSITY OF HEALTH SCIENCES, ANKARA TRAINING AND RESEARCH HOSPITAL, ANKARA, TURKEY|İZMIR BAKIRÇAY UNIVERSITY ÇIĞLI TRAINING AND RESEARCH HOSPITAL, İZMIR, TURKEY|BURSA CITY HOSPITAL, BURSA, TURKEY|TINAZTEPE HOSPITAL, İZMIR, TURKEY|ANKARA UNIVERSITY CHILDRENS'S HOSPITAL, ANKARA, TURKEY|NECMETTIN ERBAKAN UNIVERSITY MERAM FACULTY OF MEDICINE, KONYA, TURKEY|UNIVERSITY OF HEALTH SCIENCES IZMIR TEPECIK TRAINING HOSPITAL, IZMIR, TURKEY|DR.SAMI ULUS MATERNITY AND CHILDREN'S EDUCATION AND RESEARCH HOSPITAL, ANKARA, TURKEY|ORDU EDUCATION AND RESEARCH HOSPITAL, ORDU, TURKEY|UNIVERSITY OF HEALTH SCIENCES ISTANBUL KANUNI SULTAN SULEYMAN TRAINING AND RESEARCH HOSPITAL, ISTANBUL, TURKEY|ANKARA CITY HOSPITAL, ANKARA, TURKEY|KOCAELI UNIVERCITY MEDICAL FACULTY, KOCAELI, TURKEY|ISTANBUL UNIVERSITY-CERRAHPASA, CERRAHPASA FACULTY OF MEDICINE, ISTANBUL, TURKEY|HASEKI TRAINING AND RESEARCH HOSPITAL, İSTANBUL, TURKEY|HAFSA SULTAN HOSPITAL, MANISA, TURKEY|19 MAYIS UNIVERSITY, SAMSUN, TURKEY|KONYA CITY HOSPITAL, KARATAY, TURKEY|SISLI HAMIDIYE ETFAL TRAINING AND RESEARCH HOSPITAL, ISTANBUL, TURKEY|TRAKYA UNIVERSITY HOSPITAL, EDIRNE, TURKEY|MARMARA UNIVERSITY PENDIK RESEARCH AND TRAINING HOSPITAL, ISTANBUL, TURKEY|KOC UNIVERSITY HOSPITAL, ISTANBUL, TURKEY|ATATÜRK UNIVERSITY, ERZURUM, TURKEY|HACETTEPE UNIVERSITY IHSAN DOĞRAMACI CHILDREN'S HOSPITAL, ANKARA, TURKEY|TOBBETU SCHOOL OF MEDICINE, ÇANKAYA, TURKEY |BIRUNI UNIVERSITY HOSPITAL, ISTANBUL, TURKEY |ISTANBUL UNIVERSITY, ISTANBUL FACULTY OF MEDICINE HOSPITAL, ISTANBUL, TURKEY |MERSIN UNIVERSITY HOSPITAL, MERSIN, TURKEY |DOKUZ EYLUL UNIVERSITY, İZMIR, TURKEY |BEYKENT UNIVERSITY MEDICAL FACULTY, KÜÇÜKÇEKMECE, TURKEY |ETLIK ZUBEYDE HANIM WOMEN'S HEALTH TEACHING AND RESEARCH HOSPITAL,

ANKARA, TURKEY |SIVAS CUMHURIYET UNIVERSITY FACULTY OF MEDICINE, SIVAS, TURKEY, SIVAS, TURKEY |ANKARA ATATÜRK SANATORYUM TRAİNİNG AND RESEARCH HOSPİTAL, KEÇİÖREN, TURKEY |AKDENIZ UNIVERSITY, ANTALYA, TURKEY |AYDIN ADNAN MENDERES UNIVERSITY, AYDIN, TURKEY |UNIVERSITY OF HEALTH SCIENCES, GÜLHANE TRAINING AND RESEARCH HOSPITAL, ANKARA, TURKEY |EGE UNIVERSITY HOSPITAL, BORNOVA, TURKEY
